# Exploring the human small intestinal luminal microbiome via a newly developed ingestible sampling device

**DOI:** 10.1093/ismeco/ycaf224

**Published:** 2025-11-28

**Authors:** Alexandre Tronel, Morgane Roger-Margueritat, Caroline Plazy, Salomé Biennier, Anthony Craspay, Ipsita Mohanty, Stéphanie Cools Portier, Manolo Laiola, Guus Roeselers, Nicolas Mathieu, Marianne Hupe, Pieter C Dorrestein, Jean-Pierre Alcaraz, Donald Martin, Philippe Cinquin, Anne-Sophie Silvent, Joris Giai, Marion Proust, Thomas Soranzo, Elena Buelow, Audrey Le Gouellec

**Affiliations:** Pelican Health, 5 avenue du Grand Sablon, La Tronche 38700, France; Univ. Grenoble Alpes, CNRS, UMR 5525, VetAgro Sup, Grenoble INP, CHU Grenoble Alpes, TIMC, Grenoble 38000, France; Univ. Grenoble Alpes, CNRS, UMR 5525, VetAgro Sup, Grenoble INP, CHU Grenoble Alpes, TIMC, Grenoble 38000, France; Univ. Grenoble Alpes, CNRS, UMR 5525, VetAgro Sup, Grenoble INP, CHU Grenoble Alpes, TIMC, Grenoble 38000, France; Service de Biochimie Biologie Moléculaire Toxicologie Environnementale, UM Biochimie des Enzymes et des Protéines, Institut de Biologie et Pathologie, CHU Grenoble-Alpes, Grenoble 38000, France; Plateforme de Métabolomique GEMELI-GExiM, Institut de Biologie et Pathologie, CHU Grenoble-Alpes, Grenoble 38000, France; Pelican Health, 5 avenue du Grand Sablon, La Tronche 38700, France; Pelican Health, 5 avenue du Grand Sablon, La Tronche 38700, France; Skaggs School of Pharmacy and Pharmaceutical Sciences, University of California San Diego, La Jolla, CA 92093-0657, United States; Danone Global Research & Innovation Center, Route départementale 128, 91190, Gif sur Yvette, France; Danone Global Research & Innovation Center, Route départementale 128, 91190, Gif sur Yvette, France; Danone Global Research & Innovation Center, Route départementale 128, 91190, Gif sur Yvette, France; Univ. Grenoble Alpes/Hepato-Gastroenterology and Digestive Oncology Department, CHU Grenoble Alpes/Institute for Advanced Biosciences, CNRS UMR 5309-INSERM U1209, Grenoble 38043, France; Univ. Grenoble Alpes/Hepato-Gastroenterology and Digestive Oncology Department, CHU Grenoble Alpes/Institute for Advanced Biosciences, CNRS UMR 5309-INSERM U1209, Grenoble 38043, France; Skaggs School of Pharmacy and Pharmaceutical Sciences, University of California San Diego, La Jolla, CA 92093-0657, United States; Center for Microbiome Innovation, University of California San Diego, La Jolla, CA 92093, United States; Department of Pharmacology, University of California San Diego, La Jolla, CA 92093, United States; Collaborative Mass Spectrometry Innovation Center, Skaggs School of Pharmacy and Pharmaceutical Sciences, University of California San Diego, La Jolla, CA 92093-0657, United States; Univ. Grenoble Alpes, CNRS, UMR 5525, VetAgro Sup, Grenoble INP, CHU Grenoble Alpes, TIMC, Grenoble 38000, France; Univ. Grenoble Alpes, CNRS, UMR 5525, VetAgro Sup, Grenoble INP, CHU Grenoble Alpes, TIMC, Grenoble 38000, France; Univ. Grenoble Alpes, CNRS, UMR 5525, VetAgro Sup, Grenoble INP, CHU Grenoble Alpes, TIMC, Grenoble 38000, France; CIC, Univ. Grenoble Alpes, Inserm, CHU Grenoble Alpes, Grenoble 38000, France; Univ. Grenoble Alpes, CNRS, UMR 5525, VetAgro Sup, Grenoble INP, CHU Grenoble Alpes, TIMC, Grenoble 38000, France; CIC, Univ. Grenoble Alpes, Inserm, CHU Grenoble Alpes, Grenoble 38000, France; CIC, Univ. Grenoble Alpes, Inserm, CHU Grenoble Alpes, Grenoble 38000, France; Pelican Health, 5 avenue du Grand Sablon, La Tronche 38700, France; Univ. Grenoble Alpes, CNRS, UMR 5525, VetAgro Sup, Grenoble INP, CHU Grenoble Alpes, TIMC, Grenoble 38000, France; Univ. Grenoble Alpes, CNRS, UMR 5525, VetAgro Sup, Grenoble INP, CHU Grenoble Alpes, TIMC, Grenoble 38000, France; Service de Biochimie Biologie Moléculaire Toxicologie Environnementale, UM Biochimie des Enzymes et des Protéines, Institut de Biologie et Pathologie, CHU Grenoble-Alpes, Grenoble 38000, France; Plateforme de Métabolomique GEMELI-GExiM, Institut de Biologie et Pathologie, CHU Grenoble-Alpes, Grenoble 38000, France

**Keywords:** small intestine, microbiome, multi-omics, sampling medical device, healthy subjects

## Abstract

Because accessing the small intestine is technically challenging, studies of the small intestinal microbiome are predominantly conducted in patients rather than in healthy individuals. Invasive clinical procedures, such as endoscopy or surgery, usually performed for therapeutic purposes, are typically required for sample collection. Although stomas offer a less invasive means for repeated sampling, their use remains restricted to patient populations. As a result, the small intestinal microbiome of healthy individuals remains largely understudied. This study evaluated a novel ingestible medical device for collecting luminal samples from the small intestine. A monocentric interventional trial (NCT05477069) was conducted on 15 healthy subjects. Metagenomics, metabolomics, and culturomics were used to assess the effectiveness of the medical device in characterizing the healthy small intestinal microbiome and identifying potential biomarkers. The small intestinal microbiota differed significantly from the fecal microbiota, displaying high inter-individual variability, lower species richness and reduced alpha diversity. A combined untargeted and semi-targeted LC–MS/MS metabolomics approach identified a distinct small intestinal metabolic footprint, with bile acids and amino acids being the most abundant metabolite classes. Host- and host/microbe-derived bile acids were particularly abundant in small intestinal samples. Using a fast culturomics approach on two small intestinal samples, we achieved species-level characterization and identified 90 bacterial species, including five potentially novel ones. This study demonstrates the efficacy of our novel sampling device in enabling comprehensive small intestinal microbiome analysis through an integrative, multi-omics approach. This approach allows distinct microbiome signatures to be identified between small intestinal and fecal samples.

## Graphical Abstract

**Graphical Abstract:**
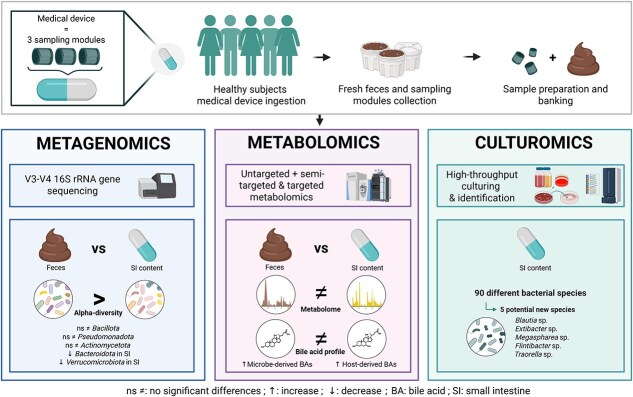

## Introduction

The small intestine (SI) plays a pivotal role in digestion, nutrient absorption, and host–microbiota interactions, yet its microbial ecosystem remains poorly characterized, particularly in healthy individuals. The gut microbiota as a whole constitutes a complex ecosystem of bacteria, fungi, viruses, protists, and archaea that directly supports gut homeostasis and host health through key functions such as metabolism [1], immune regulation [2], endocrine regulation [3, 4], and regulation of organ physiology (e.g., gut-brain [5] and gut-liver axes) [6]. Microbial, host-, and diet-derived metabolites mediate both microbe–microbe and host–microbe interactions. Environmental factors—such as oxygen [7], pH [8], mucus [9], and host secretions [10] (pancreatic juice, bile, saliva, gastric juice)—strongly shape microbiota composition along the gastrointestinal tract (GIT). Oxygen levels for instance decrease progressively along the GIT, creating an anoxic environment in the distal intestine [11]. Transit time and substrate availability also influence microbiota [12]; the SI is a more dynamic niche with a shorter transit time (2–5 hours transit) than the colon [13–15]. In the SI, bile acids (BAs), produced by hepatocytes, facilitate hydrophobic compounds absorption such as lipids [16, 17]. Beyond this role, BAs modify the gut microbiota by inducing damage to the bacterial membrane through their surfactant properties and can cause DNA damage and oxidative stress on cells [18].

Consequently, bacterial density and diversity increase from the proximal (10^3^ CFU/mL) to the distal (10^11^ CFU/mL) parts of the gut [19], with microbial composition directly influencing the gut functionalities. The SI microbiota plays a key role in the biotransformation of BAs, notably through bile salt hydrolases (BSHs) that deconjugate conjugated BAs, releasing free molecules [10, 20, 21]. Evidence also indicates that SI microbiota is enriched in genes for carbohydrate transport, central metabolism, and biotin production compared with fecal microbiota [12].

However, most microbiome studies have focused on fecal samples, which largely reflect the distal gut, leaving the SI microbiota understudied [22, 23]. Access to this region is limited, and most investigations have relied on invasive sampling via surgery [24], endoscopy [25, 26], ileostomy [12, 27–30], or catheter aspiration [31]. These methods pose several challenges, including possible contamination [32], microbiota modifications caused by intestinal preparation [33, 34], and ethical constraints in healthy population. Given the SI’s central physiological role, it is imperative to develop new, simple, and non-invasive strategies for intestinal sampling. The development of medical devices (MD) capable of non-invasively collecting SI offers a promising approach to overcoming these challenges [35]. To date, several devices have been described, and some clinical investigations have evaluated their performance [36–39] and demonstrated that the SI microbiome is less diverse than the fecal microbiome. The SI microbiota is known to be dominated by the genera *Lactobacillus, Clostridium, Streptococcus, Staphylococcus, Veillonella* and *Bacteroides* [22–24, 40–43]. However, the bacterial groups identified in the SI are predominantly characterized using metagenomic approaches, which overlook the cellular viability of the described species. Culturomics-based methods can bridge this gap, yet only a few studies have employed them to date [36, 42, 44]. Culturomics is a powerful complementary approach that enables species discovery and strain level characterization, with important implications for the development of therapeutic probiotics [45]. It enhances metagenomic analysis by capturing low abundance species that otherwise may escape sequencing detection [46]. Despite its significance, the metabolome of the SI also remains poorly characterized. Most studies have relied on targeted or non-targeted metabolomics focused solely on short-chain fatty acids (SCFAs) and BAs [36, 47, 48], thereby providing an incomplete view of the complex metabolic interactions shaping this unique environment.

In this study, a first in man clinical investigation was conducted (detailed protocol described elsewhere [37]) to evaluate the safety and performance of a novel sampling MD that collects intestinal content from the SI in a non-invasive manner. The technology utilizes a pH-dependent enteric capsule and is the only equipped with three sampling modules, enabling multi-sampling within the SI. The secondary objectives of the study were to perform multi-omics analysis to characterize the upper intestinal microbiota composition and metabolome with a focus on the identification of potential biomarkers specific for the SI. In this study, we initially demonstrated the feasibility of conducting multi-omic analyses (metagenomics, metabolomics and culturomics) on SI contents. By analyzing the corresponding feces, we demonstrated significant differences in microbial composition and metabolites between SI contents and fecal samples. The analysis of SI contents revealed the presence of specific metabolites, including glycocholic acid and taurocholic acid, which are host-derived BAs. Furthermore, culturomics was employed to characterize and archive 90 bacterial species including five previously uncharacterized species. This comprehensive approach underscores the efficacy of the MD for elucidating the intricate composition of the SI microbiome in healthy individuals and for the prospective identification of novel biological markers.

## Materials and methods

### Description of the sampling medical device

The sampling MD employed in this clinical investigation was developed by Pelican Health SAS, La Tronche, France (patent number: WO2019081539) [49]. The device is designed for single-use, is ingestible and is minimally invasive. It is intended for the collection of samples from the luminal small intestinal fluid. The device consists of a gastro-resistant and pH-dependent pill (size 00) for enteric use and three sampling modules with a final size of 8 mm x 22 mm (Supplementary Fig. 1). Each sampling module is composed of silicone and contains a compressed super-absorbent polymer (comparable to a sponge) that functions as a reservoir. The size of a sampling module is 6.5 mm x 7 mm. Upon the introduction of fluid into the module, the compressed polymer undergoes expansion, resulting in the release of mechanical energy that activates the closing of valves. This process effectively isolates the sample from the external environment. The complete modules are then transferred through the large bowel and subsequently collected in the feces for further analysis.

### Participants and study design

The clinical investigation protocol has been previously described in more detail [37]. The main inclusion criteria for healthy volunteers were: subjects must (1) be aged between 18 and 65 years; (2) have a body mass index (BMI) greater than 20 and lower than 30 and (3) be affiliated with a social security system. A large number of exclusion criteria were implemented to ensure that the subjects had no gastro-intestinal issues or any co-morbidities [37]. In addition, candidates taking medication (antibiotics) or probiotics and related substances were excluded. The protocol was approved by the Personal Protection Committee (CPP) and the French National Agency for the Safety of Medicines and Health Products (ANSM).

A total of 15 healthy volunteers were included during the study and each subject swallowed one MD under the supervision of a gastroenterologist following a 10 hour fast. Upon reaching the jejunal/ileal part of the SI [50], the pill released three sampling modules to collect luminal content. Fecal samples were collected in a designated collection device and stored in a cool box immediately after collection before being directly transferred to the Grenoble’s hospital. The global workflow for sample management is described in more detail in the published protocol [37]. As no modules were detected in the feces of one volunteer, only 14 SI and fecal samples were analyzed.

### Sample preparation for multi-omics analysis

Following collection, fecal samples were directly processed at the hospital. Initially, the feces were screened for the presence of the collection modules released from the MD. In the absence of a detected module, the subject’s fecal collection was continued. Conversely, if at least one module was detected in the collected fecal sample, the modules were recovered and separately processed to assess the volume sampled. The upper intestinal content was then retrieved by means of centrifugation. The bacterial pellets were subsequently utilized for metabarcoding sequencing and the residual fluids were allocated for metabolomics analysis. Concurrently, fecal samples were also processed to perform the same analysis (metabarcoding and metabolomics respectively). Additionally, for only two subjects, a culturomics approach was performed on intestinal content. All samples were stored at −80°C until analysis, except for the culturomics samples.

### Multi-omics analyses

Fecal samples and small intestinal (SI) contents were characterized using a combination of 16S rRNA gene metabarcoding (V3–V4 regions) and metabolomics to assess the bacterial composition and metabolome, respectively. Statistical analyses of 16S rRNA gene metabarcoding were performed using R software with *vegan* package [51] for diversity analysis and *phyloseq* [52] for microbiome data handling. Metabolomic profiling was performed using Liquid Chromatography–High Resolution Mass Spectrometry (LC-HRMS/MS), combining untargeted and semi-targeted approaches to define the global metabolome, as previously described in [53]. For the semi-targeted analysis, metabolites were quantified using external, multi-point calibration curves with authentic standards (covering the expected concentration ranges), and fitted using weighted linear regression. This enabled the identification of novel metabolites and the consistent quantification of 37 pre-selected metabolites [53] (Supplementary Table 1). Targeted metabolomics was also conducted to quantify 50 bile acids (BAs) (Supplementary Table 2). Additionally, a fast culturomics protocol was applied to two SI content samples. Microorganisms were cultivated on a range of solid and liquid media, then identified using Matrix-Assisted Laser Desorption/Ionization Time-of-Flight (MALDI-TOF) mass spectrometry and Sanger sequencing of the full-length 16S rRNA gene (Supplementary Fig. 2). Detailed protocols for all analyses are provided in the Supplementary Methods.

## Results

### Summary of clinical outcomes following use of the medical device

Fifteen healthy subjects have been included in the investigation (3 females/12 males) with a mean age of 29.7 and a BMI of 21.1. The safety for the use of the device was validated for all subjects. Two minor adverse events have been noticed with only one related to gastrointestinal disorders (abdominal distension). Regarding acceptance of the device, all subjects felt that their physical ability was not impaired during the study. Ingestion of the device, use of the stool collector and stool collection, as well as management of the follow-up documents, were considered easy (or even very easy) by all subjects (Table 1). Finally, the mean time to recover all modules was 40 hours, ranging from a fastest time of 23 hours to a slowest time of 149 hours (Table 1).

**Table 1:** Description of the healthy cohort and clinical outcomes.

**Cohort description and clinical outcomes**	
**Sex**	
Female (%)	3 (20%)
Male (%)	12 (80%)
**Age (year)**	
Mean (SD)	29.7 (6.8)
Median	29
Q1-Q3	24.5–34.0
Min-Max	21.0–44.0
**Body mass index (BMI)**	
Mean (SD)	21.1 (1.1)
Median	21.0
Q1-Q3	20.1–22.1
Min-Max	19.7–22.8
**Safety**	
Adverse event	2 (1 abdominal distension, 1 ligament sprain)
Adverse effect	0
**Acceptability**	
No alteration of physical capacity	15
Ease of use	15 (simple/very simple to use)
**Module recovery**	
**Time** (hour)	
Mean (SD)	40 (21.7)
Median	33
Q1-Q3	24–48
Min-Max	23–149
**Total recovery** (%)	13 (87%)

(SD): Standard deviation. Q1-Q3: first and third quartile, Min-Max: minimal and maximal value, n = 15

Utilizing our MD, we obtained samples of SI content and fecal samples from 14 healthy volunteers and compared their microbiota and metabolic profiles by metagenomic barcoding, culturomics and metabolomics.

### The SI microbiota differs from the fecal microbiome

**Figure 1:**
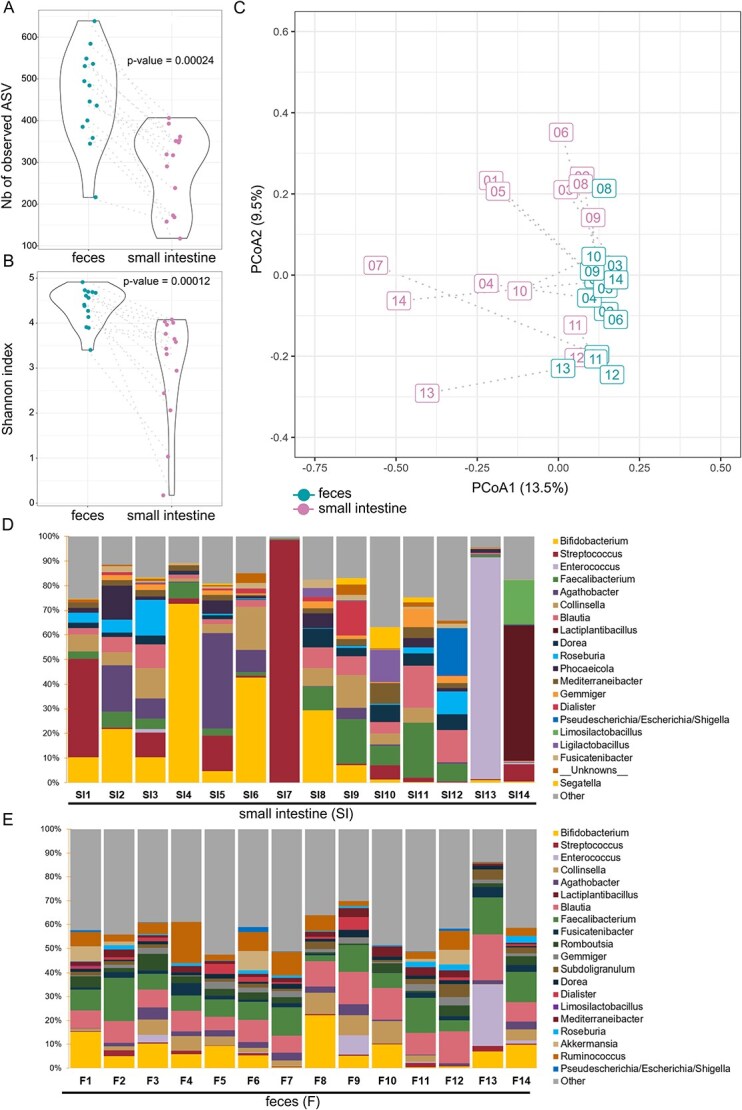
Microbial composition of the small intestinal contents (n = 14) and fecal samples (n = 14). (A) Bacterial richness and (B) bacterial diversity (Shannon index) in SI contents and fecal samples. The samples (SI content vs feces) for each subject are connected by dotted lines. (C) Microbial community β-diversity at ASV level, which was demonstrated using principal coordinates analysis (PCoA) of the bray-Curtis distance matrix. The samples (SI content vs feces) for each subject are connected by dotted lines. (D) Relative abundance of the 20 most predominant bacterial genera in small intestinal contents. Remaining genera are summarized as “other”. Each column represents one subject. (E) Relative abundance of the 20 most abundant bacterial genera in fecal samples. Other genera are summarized as “other”. Each column represents one subject.

Alpha-diversity metrics were calculated after normalization of all samples by the rarefaction method using the phyloseq R package [54], with a minimum read depth of 46 557 reads per sample (excluding PBS controls). A significant difference in species richness (p-value <0.001) was observed between the two sample types, with fecal samples exhibiting a higher richness (Fig. 1A). Microbial diversity, estimated by the Shannon index, also differed significantly between sample types, with the SI microbiota displaying lower diversity than fecal microbiota (p-value <0.001; Fig. 1B). Principal coordinate analysis (PCoA) on ASV level (Bray-Curtis distances) indicated significant differences in microbial composition between SI content and feces (Fig. 1C). The PERMDISP test revealed a greater dispersion within the SI content group compared to the fecal samples (p-value <0.001). This finding indicates that the SI content group exhibited higher variability, suggesting the presence of greater inter-individual variation in the upper intestinal tract (Supplementary Fig. 3).

*Bacillota* was the most abundant phylum in both sample type with a common mean relative abundance of 70%. Significant differences between sample type were only found for *Bacteroidota* (p-value <0.01) and *Verrucomicrobiota* (p-value <0.05) relative abundances (Supplementary Fig. 4). Differences were observed for the mean relative abundance of *Actinomycetota*, with a higher level in SI content (21,3% vs 12,9%). At the genus level, a higher abundance of bacteria from the genera *Streptococcus* and *Bifidobacterium* was observed in the SI contents compared to the fecal samples (Fig. 1D and 1E). Figure 1D and 1E show the 20 top general genera across samples. A notable degree of inter-individual variation in SI contents was evident. This observation was particularly pronounced for individuals exhibiting a remarkably elevated relative abundance of *Streptococcus* (SI7), *Bifidobacterium* (SI4), or *Enterococcus* (SI13) in their respective samples (Fig. 1D). Overall, fecal samples showed less inter-individual variation compared to SI contents (Fig. 1C). We calculated the relative abundance fold change (FC) for each genus to identify the most discriminating genera for the respective sample types (on average). For SI content, bacteria from the genera *Schaalia* (FC = 1.24, q-value <0.05), *Gemella* (FC = 1.19, q-value <0.05) and *Granulicatella* (FC = 1.11, q-value <0.05) were the most discriminant genera (Supplementary Table 3). On the other hand, *Ruminococcus* (FC = 0.22, q-value <0.01)*, Bacteroides* (FC = 0.34, q-value <0.01)*, Romboutsia* (FC = 0.37, q-value <0.01) and *Akkermansia* (FC = 0.44, q-value <0.05) were the most discriminant genera for feces (Supplementary Table 3). These genera may serve as potential indicators of the SI and feces microbiota respectively, in healthy individuals.

### Combination of untargeted and semi-targeted metabolomics reveals significant differences between the SI and the fecal metabolome

**Figure 2:**
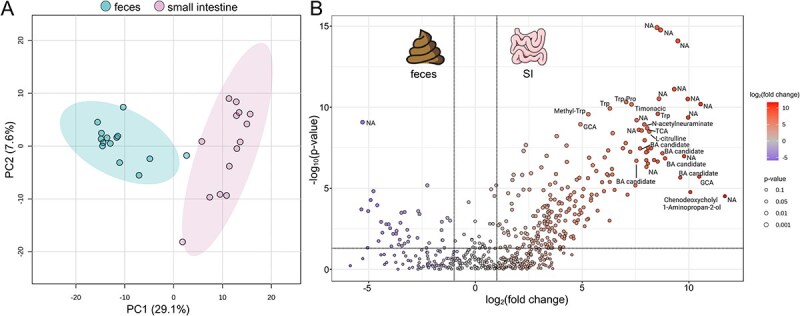
Comparison of metabolite abundance between the SI and fecal samples revealed significant differences across a wide range of compounds. (A) Principal component analysis (PCA) of the filtered features (*n* = 574 metabolites) obtained in positive ionization mode. To assess statistical differences between the metabolomics profiles, PERMANOVA based on 999 Monte Carlo permutations was performed (F-value: 45.419; R-squared: 0.63595; p-value permutations: 0.001). (B) Volcano plot of the metabolites showing significant differences in the abundance of metabolites between the SI and the feces groups using a fold change (FC) threshold of 2 and a *t*-test threshold of 0.05. The most discriminant features (top 30) have been annotated. NA: Not annotated. the log transformed FC and P-values are represented on x- and y-axes.

We also performed combined untargeted and semi-targeted metabolomics to extensively characterize the SI content versus its corresponding fecal metabolome, as previously described in [53]. In the positive ionization mode for untargeted metabolomics, we obtained 5289 features after processing with MZmine3. After filtering the data, 574 features remained, representing 10.8% of the original features. Principal component analysis (PCA) on the filtered data revealed a clear separation between SI contents and feces (p-value <0.001) (Fig. 2A). Thus, the metabolomic profiles of the SI contents differed substantially from those of the fecal samples (R-squared: 0.63595). Similar separation was observed in the PCA based on the data from negative ionization mode where 217 filtered features (representing 2.4% of the original features) were used for the analysis (Supplementary Fig. 5).

To identify abundance differences in metabolites between the two sample types, we performed supervised statistical analyses. The volcano plot based on the 574 features shows 206 features which were more abundant in SI contents (p-value <0.05) and 48 features more abundant in feces (p-value <0.05). From the 574 features, 189 could be annotated to the level 2 of annotation, meaning that the MS2 spectra matched to spectrum libraries [55] (i.e, 33%). A total of 45% of the features were annotated among those with a p-value ≤0.05 and an FC ≥ 2.115. The largest chemical annotated subclasses were amino acids/peptides and BAs (Supplementary Table 4). A differential analysis of the metabolites revealed that certain metabolites, including BAs (glycocholic acid [GCA] and taurocholic acid [TCA]), as well as some candidate BAs, and amino acids (tryptophan, methyl-tryptophan, and citrulline) exhibited higher concentrations in the SI content compared to feces (Fig. 2B).

**Figure 3:**
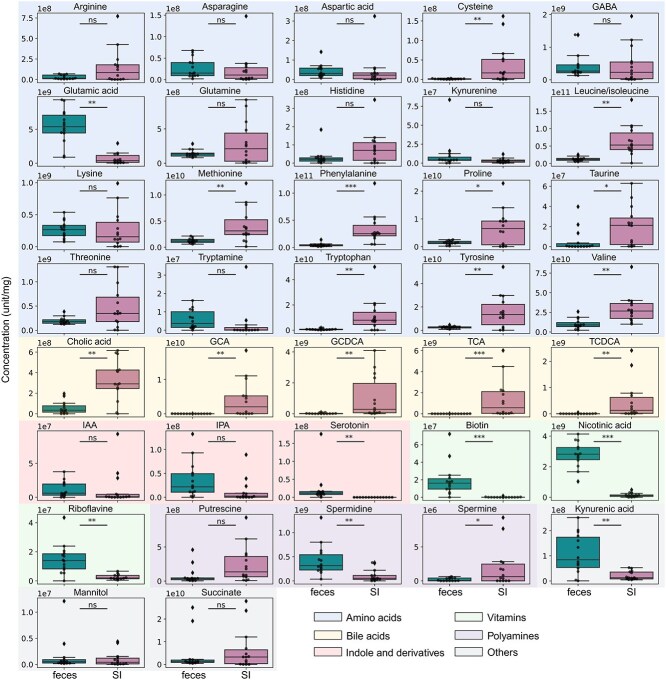
Relative quantification by semi-targeted metabolomics of the metabolites of interest in small intestinal contents (n = 14) and fecal samples (n = 14) (unit/mg). GCA: Glycocholic acid; GCDCA: Glycochenodeoxycholic acid; TCA: Taurocholic acid; TCDCA: Taurochenodeoxycholic acid; IAA: Indole-3-acetic acid; IPA: Indole-3-propoinic acid. Wilcoxon test with adjusted p-value by using the Benjamini-Hochberg procedure with the false discovery rate (FDR). ^*^: 0.01 < p-value <0.05, ^**^: 0.001 < p-value <0.01, ^***^: 0.0001 < p-value <0.001, ns: Non-significant.

For semi-targeted metabolomics, 37 metabolites were targeted in both the SI content and fecal samples. A total of 15 metabolites were found to be significantly more abundant in SI content compared to feces, particularly those derived from amino acids (tryptophan, phenylalanine, and leucine/isoleucine) and from the BA group (Fig. 3). Conversely, seven metabolites were significantly more abundant in feces compared to SI content (e.g. biotin (p-value <0.001), nicotinic acid (p-value <0.001) and serotonin (p-value <0.01)). For instance, the mean concentration of tryptophan in SI content was estimated to be 1.15 x 10^10^ unit/μL and 7.16 x 10^8^ unit/mg in feces, and the mean concentration of nicotinic acid (vitamin B3) was estimated to be 1.39 x 10^8^ unit/μL and 2.78 x 10^9^ unit/mg in feces (Fig. 3). These findings suggest that these concentrations may be indicative of their average concentration in healthy individuals.

### The SI microbiome: A significant reservoir for BAs

**Figure 4:**
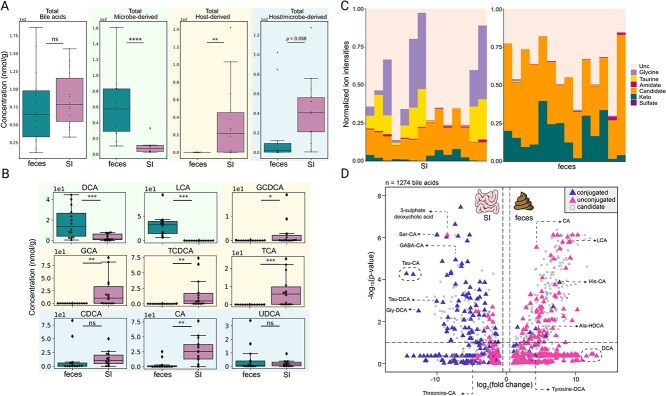
Determination of the bile acids (BAs) profile in SI contents compared to their corresponding fecal samples. (A) Boxplots of the overall concentration of BAs in SI (n = 14) and fecal (n = 14) samples. Concentration of total microbe-derived BAs and total host-derived BAs were depicted and compared separately as well as total host/microbe-derived BAs (nmol/g). (B) Boxplots of the most common BAs quantified in SI content (n = 14) or fecal sample (n = 14). Microbe-derived BAs colored in green, host-derived BAs colored in yellow and host/microbe-derived BAs colored in blue. DCA: Deoxycholic acid; LCA: Lithocholic acid; GCDCA: Glycochenodeoxycholic acid; GCA: Glycocholic acid; TCDCA: Taurochenodeoxycholic acid; TCA: Taurocholic acid; CDCA: Chenodeoxycholic acid; CA: Cholic acid and UDCA: Ursodeoxycholic acid. (C) Global profile of bile acids in SI contents and fecal samples. Each column represents a given sample from one subject. BAs have been grouped according to their chemical family, Unc: Unconjugated BA. (D) Volcano plot demonstrating the log_2_ fold change in the peak area abundances of the 14 subjects between their SI contents and fecal samples. Wilcoxon test with adjusted p-value by using the Benjamini-Hochberg procedure with the false discovery rate (FDR). ^*^: 0.01 < p-value <0.05, ^**^: 0.001 < p-value <0.01, ^***^: 0.0001 < p-value <0.001, ^****^: p-value <0.0001, ns: Non-significant.

The application of targeted metabolomics on BAs revealed no statistically significant disparities between the SI content and the corresponding fecal samples, with respect to all targeted BAs (50 BAs). However, a significant difference was identified between the SI content and feces when BAs were categorized based on their source (host vs. microbe-derived BAs, respectively) (Fig. 4A). The analysis revealed that microbe-derived BAs exhibited a marked increase in concentration in fecal samples (p-value <0.0001), while host-derived BAs demonstrated a significantly higher concentration in the SI content (p-value <0.01). Furthermore, BAs that can derive from both the host and microbes (host/microbe-derived BAs) were found to be more abundant in the SI content (p-value <0.058) (Fig. 4A). The absolute concentrations for 50 BAs were subsequently determined (Fig. 4B and Supplementary Fig. 6). The mean concentrations of primary deconjugated BAs, cholic acid (CA) and chenodeoxycholic acid (CDCA), were evaluated to be 27.5 μM ± 21.6 μM and 13.9 μM ± 13.4 μM in the SI contents and 3.5 μM ± 7.6 μM and 13.4 μM ± 26.8 μM in feces, respectively. For glycine and taurine-conjugated BAs, the mean concentrations of GCA, glycochenodeoxycholic acid (GCDCA), TCA, and taurochenodeoxycholic acid (TCDCA) were found to be significantly higher in the SI contents compared to feces (23.7 μM ± 31.4 μM in SI contents versus 0.02 μM ± 0.02 μM in feces, 3.9 μM ± 6.2 μM in SI contents versus lower the limit of detection in feces, 7.4 μM ± 8.7 μM in SI contents versus 0.02 μM ± 0.03 μM in feces, and 1.6 μM ± 2.4 μM in SI contents versus 0.02 μM ± 0.02 μM in feces, respectively) (Fig. 4B). Additionally, the results obtained for the BAs detected using both the semi-targeted and targeted approaches revealed similar abundance trends and levels of significance.

Conversely, secondary BAs were more abundant in feces. For example, deoxycholic acid (DCA) and lithocholic acid (LCA) mean concentrations were measured at 3.44 μM ± 3.1 μM in SI contents versus 17.3 μM ± 14.8 μM in feces and 0.2 μM ± 0.1 μM in SI contents versus 30.5 μM ± 22.4 μM in feces, respectively. The present study offers an initial overview of BA concentrations in healthy subjects (Supplementary Table 5). We determined that the median host-derived BA concentration was 21.56 μM in SI samples and 0.01 μM in feces.

The global BA profile of each sample was determined using an untargeted metabolomics approach. Distinct profiles were identified between the two sample types, with a higher abundance of glycine and taurine conjugated BAs in SI content (Fig. 4C). Conversely, fecal samples exhibited a higher abundance of Keto-BAs and candidate BAs derived from a novel BA library [56]. The analysis revealed interindividual variations within SI contents and feces groups, with five subjects exhibiting remarkably low abundances of glycine and taurine conjugated BAs in their SI contents. Utilizing the untargeted data, we observed a robust cluster of unconjugated BAs that exhibited higher levels in feces compared to the conjugated BAs present in the SI (Fig. 4D). However, there were a few exceptions, including the histidine and alanine conjugated BAs, which appeared to be elevated in feces. Furthermore, sulphated deoxycholic acid levels were found to be elevated in the SI. We performed a molecular network analysis on BAs using FBMN on GNPS2 using the untargeted data (Supplementary Fig. 7). MS2 spectral matches to 68 BAs were obtained by matching to the BILELIB19 library, which increased to 556 annotations by matching to the recently generated candidate bile acid library [56]. Matches to amino acid and other amine conjugated BAs were also recovered, with varying distribution between SI contents and feces.

### Characterization of the small intestinal content by culturomics

**Figure 5:**
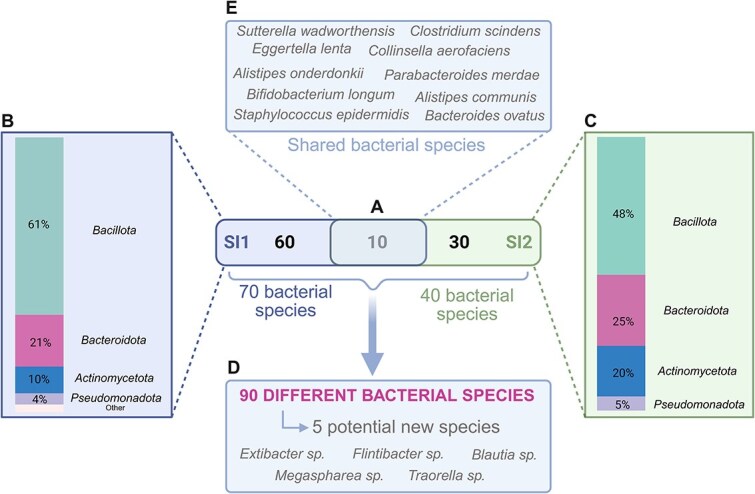
Isolated bacterial species by means of culturomics for small intestinal contents sampled from two different healthy volunteers. (A) Total number of microbial species identified for each sample. SI1: SI content 1 and SI2: SI content 2. (B) Percentage of identified species for each bacterial phylum for the SI content 1. “Other” contains two phyla: *Thermodesulfobacteriota* (1,4%) and *Campylobacterota* (1,4%). (C) Percentage of identified species for each bacterial phylum for the SI content 2. (D) Total number of distinct microbial species identified during both culturomics and including the number of the discovered new bacterial species. (E) Core bacterial species identified in both SI contents (figure created in BioRender).

The SI microbiota has not yet been the subject of culturomic study, a culture approach that involves the cultivation of as many bacterial species as possible through the use of enrichment steps, specific media, and incubation periods under both aerobic and anaerobic conditions. The culture-based analysis of the SI microbiota has the potential to facilitate the discovery of new species and the extensive study of isolated human gut bacteria that could have important implications in health and disease. Two SI contents recovered from two different healthy subjects were analyzed by culturomics. For the SI content of the first healthy subject, 733 colonies were isolated (666 in anaerobic and 67 in aerobic culture conditions). Subsequent identification of the isolates was facilitated by MALDI-TOF, resulting in precise species designation for 89% of the isolated strains. The remaining 11% of the isolates were identified through the sequencing of the entire 16S rRNA gene. The collective analysis yielded the identification of 70 distinct bacterial species (see Fig. 5A and refer to the data in Supplementary Table 6). The majority of these isolates were classified into the phyla *Bacillota* and *Bacteroidota*, accounting for 60% and 21% of the identified species, respectively (refer to Fig. 5B for further details). A total of four potential novel bacterial species belonging to the genera *Blautia, Extibacter, Flintibacter* and *Megaspharea* were identified based on their full-length 16S rRNA gene sequences. Their nucleotide similarities were less than 98.65% compared to publicly available and annotated 16S rRNA gene databases (EZbiocloud database update 2023.08.23) (see Fig. 5D). The evaluation of the number of isolates and the final number of potentially newly identified species revealed the presence of one species for every 10.4 isolations. A second SI content was obtained from a different healthy subject and analyzed by culturomics. Overall, 736 colonies were isolated (716 in anaerobic and 20 in aerobic culture conditions). A total of 87% of the isolates could be identified by MALDI-TOF, leading to the identification of 40 bacterial species (Fig. 5A and Supplementary Table 6). The majority of the 40 isolated bacterial species belonged to the phylum *Bacillota* and *Bacteroidota* (48% and 25% respectively; Fig. 5C). For the SI content of this second healthy individual, we identified one potential new bacterial species belonging to the genus *Traorella* (Fig. 5D)*.* Combining both samples yielded a total of 90 unique bacterial species isolated and preserved during the course of the experiments (Fig. 5D). Ten bacterial species were found in both samples (Fig. 5E). Detailed growth conditions for each species are described in Supplementary Table 7. A comparison of the bacterial genera identified during culturomics with those detected by 16S metabarcoding revealed that 27 genera were exclusively detected by culturomics in sample 1 and 11 genera were identified in sample 2 (Supplementary Table 8). Conversely, 40 and 39 genera were exclusively identified through 16S metabarcoding in sample 1 and 2 respectively.

## Discussion

The SI is a key section of the GIT where essential process such as digestion and nutrient absorption occurs. Unlike the fecal microbiome, which has been extensively studied and better characterized, the SI microbiome remains largely underexplored due to the difficulty of accessing sample. Indeed, the main challenge for studying the SI microbiome is its accessibility, usually requiring invasive methods to obtain SI content samples. Here, we investigated the microbial and metabolite composition of SI contents sampled with a new sampling MD based on pH-dependent release technology, compared to corresponding fecal samples in 14 healthy subjects. It is important to note that inter-individual variations in gastrointestinal pH may influence the precise sampling location of the MD [57–59]. Furthermore, validation of sampling sites remains challenging, as current reference methods are highly invasive and not suitable for use in healthy individuals. Nevertheless, we aimed to show that the new MD could significantly advance the characterization of the SI microbiome of healthy volunteers and that its future application in SI microbiome studies and the design of clinical trials will aid to eventually identify new diagnostic biomarkers of disease associated with the SI microbiome.

Our results demonstrate that the microbial composition of the SI differed significantly from that of the fecal samples, with a lower ASVs richness and α-diversity in the SI. Gram-positive genera such as *Schaalia*, *Gemella*, *Granulicatella* were determined as discriminant taxa for SI microbiota (Supplementary Table 3). A comparison was made between microbial outcomes obtained in this study and those from multiple preceding investigations highlighting that the SI microbiota is less diverse than fecal microbiota (Supplementary Table 9). The results of microbial composition vary depending on the study, but the majority of studies show a predominance of facultative anaerobic bacteria such as *Streptococcus*. Some studies have demonstrated relatively high abundances of *Enterobacteriaceae* especially *Escherichia*/*Shigella* [27, 28, 36] (Supplementary Table 9). In the study, we also detected a trend that the relative abundance of *Streptococcus*, *Veillonella* and *Prevotella* was greater in SI contents compared to feces. It is imperative to note that a significant degree of inter-individual variation was detected within the SI microbiota group. Higher abundance of *Bifidobacterium* and *Faecalibacterium* was observed in some subjects of the SI content group compared to other subjects in our study (Fig. 1D) and to the others clinical studies describing the SI microbiota [31, 36, 38, 39]. The observed variations could be attributed to disparities in the designated sites of collection. Indeed, the MD is pH-sensitive and disintegrates at enteric pH [50]. Possible inter-individual pH variations [60], buffer capacity [61–63], intestinal fluid quantities and repartitions [64] could influence the location where the MD disintegrates and therefore the SI sample collection site. Furthermore, it is plausible that the bacterial composition within the sampling module undergoes alterations during transit, as the MD is devoid of preservatives that would stabilize the bacterial composition. Consequently, the continued proliferation of certain bacterial species during transit has the potential to distort the microbial composition of the collection site. Moreover, baseline inter-individual differences in microbiome composition may further contribute to the variability observed across samples, complicating the distinction between biological variation and sampling-related effects. Procházková et al., have recently shown the impact of gut physiology and the environment on the composition and metabolism of the gut microbiome, with the pH and transit time of each volunteer affecting the microbiome composition [59].

To achieve an unprecedented characterisation of SI metabolome in healthy volunteers, we applied a combined approach integrating untargeted and semi-targeted metabolomics [53]. This strategy allowed us to determine the global metabolic profiles of SI contents and fecal samples, highlighting a distinct metabolic signature specific to each sample type. We identified key metabolites that were unique or significantly more abundant in one sample type compared to other, including TCA, CGA, and CA. Notably, BAs emerged as major discriminant metabolite identified through untargeted metabolomics, explaining many of the differences between the sample types. Using the semi-targeted metabolomics, we quantified 37 key metabolites and found that 15 of them were significantly more abundant in SI contents, with a large proportion corresponding to amino acids and BAs. The high quantity of BAs and amino acids observed in the SI contents likely reflect the intestine’s primary functions, such as digestion and nutrient absorption [31, 48]. This finding aligns with previous report by Folz et al., who also described elevated levels of BAs and protein breakdown products in the SI [48]. Similar trends were observed by Wang et al., where cysteine, histidine and arginine were more abundant in SI contents compared to feces (Fig. 3) [39]. Amino acids, which are derived from the degradation of proteins and peptides, are absorbed predominantly in the SI, while primary BAs, secreted by the host in the duodenum, play a major role in emulsifying lipids and facilitating their absorption [65]. These functional roles explain their relatively high abundance in the SI microbiota. The results obtained during the characterization of BA profile are similar to those found in previous studies, where concentrations of conjugated and unconjugated primary BAs are higher in SI contents [36, 39]. Similarly to Shalon et al., a higher abundance of Ser-trihydroxylated BAs was identified in SI contents compared to feces (Fig. 4D) [36].

Consistent with our findings, we showed that microbe-derived BAs were significantly more abundant in fecal samples, while host-derived BAs were predominantly concentrated in SI contents. Additionally, host/microbe-derived BAs appeared to be more abundant in SI contents, although this difference was only marginally significant (p-value = 0.058), possibly due to intra-individual variations and the relatively small sample size. Notably, no significant difference was detected in the total BA concentration, likely because the subjects ingested the MD after a 10-hour fast. The predominance of host-derived BAs (such as GCA, TCA) in SI contents is expected since BAs are secreted in the duodenum via bile following meals [65]. Conversely, the higher abundance of microbe-derived BAs in feces likely reflects the increased microbial load and diversity in the colon, which promotes the transformation of primary BAs into secondary BAs by microbial enzymatic activity. BA concentrations can vary significantly due to several factors including dietary habits and circadian rhythm. For example, taurine- and glycine-conjugated BAs typically peak around mealtimes, while unconjugated BAs (CA, CDCA, LCA, DCA) peak around midnight and remain stable throughout the night [66]. Additionally, age-related shifts in the BA pool have been reported, with older individuals showing higher level of conjugated BAs, particularly taurine conjugates [67]. These variations make BA profiling across the healthy population challenging and highlights the need for clinical trials using a non-invasive MD to establish a robust and comprehensive characterization of the healthy SI metabolome.

Our study also suggests that the bacterial enzyme bile salt hydrolase (BSH) is capable of deconjugating not only taurine and glycine conjugates but also other amino acid conjugates. Importantly, most BSH-expressing bacteria are predominantly present in the large intestine. Examining the distribution of predicted candidate bile acids (BAs) revealed differential abundances between small intestinal (SI) and fecal samples. BAs function as key signaling molecules and microbiome metabolites that influence host–microbe interactions. Dysregulation of BA metabolism has been associated with various diseases, including inflammatory bowel disease (IBD). In IBD, for example, alterations in BA metabolism can result in increased levels of primary BAs and decreased level of secondary BAs [68], which may contribute to the dysregulation of BA receptors like the Farnesoid X receptor (FXR). FXR plays crucial roles in regulating inflammation, autophagy, and immune responses [69]. Furthermore, the microbial transformation of BAs and their interaction with receptors have been linked to a wide range of metabolic, infectious, and neoplastic diseases [70, 71]. Understanding these interactions could open new avenues for developing therapeutic strategies targeting BAs and their receptors in gastrointestinal and systemic diseases [72].

To thoroughly characterize human SI microbial functions, a culturomics approach is useful. In this study, fast culturomics enabled the isolation and preservation of a diverse range of human SI gut bacteria, yielding a total of 90 species from two healthy subjects. Among these, we identified primarily anaerobic species, including several potentially novel microbial species (*Blautia sp.*, *Extibacter sp.*, *Flintibacter sp*., *Megasphaera sp.*, and *Traorella sp.*). By comparing the bacterial genera isolated via culturomics with those detected through 16S rRNA gene sequencing, we observed that some genera were uniquely identified by one method but not the other. In 2025, Diop et al., also described the importance of combining culturomics with metagenomics, as 94 individual species were uniquely detected by culturomics [46]. This highlights the complementary nature of these approaches, as each method carries inherent biases. While sequencing techniques often underrepresent low-abundance microorganisms due to methodological factors such as DNA extraction and primer specificity [73], culture techniques do not allow the isolation of all microbes [74]. Integrating culturomics with sequencing provide a more comprehensive view of the microbiota. One major advantage of culturomics lies in its ability to characterize isolates individually or in co-culture, enabling detailed phenotypic and genotypic characterization. This is particularly valuable for assessing the potential probiotic properties of specific bacterial strains.

Using a novel sampling MD, we successfully performed an in-depth characterization of the small intestinal microbiome in healthy volunteers. By combining 16S rRNA gene sequencing, untargeted and semi-targeted metabolomics, BA-targeted metabolomics, and culturomics, our study demonstrates the feasibility and effectiveness of multi-omics analysis using samples collected with this novel sampling MD.

In conclusion, the microbial profiles and metabolomes exhibited marked differences between SI contents and fecal samples, particularly in terms of BA composition. SI content showed higher concentrations of host-derived and host/microbe-derived BAs compared to feces. This finding underscores the unique metabolic environment of the SI and highlights the potential of the MD as a groundbreaking tool for studying the SI microbiome in clinical trials. By enabling multi-omics analyses on non-invasively collected samples, this device could transform diagnostic approaches and biomarker discovery. Notably, even within a small cohort of healthy subjects, we identified specific SI biomarkers, such as host-derived BAs, emphasizing the clinical relevance of this innovative sampling method.

## Supplementary Material

Supplementary_Figures_ISME_Comm_FINAL_ycaf224

Supplementary_Methods_ISME_Comm_FINAL_ycaf224

Supplementary_Tables_ISME_Comm_FINAL_ycaf224

Supplementary_table_9_FINAL_ycaf224

## Data Availability

Raw 16S rRNA gene sequences are available under accession number PRJEB102561. The metabolomics dataset generated in this study is available at MassIVE Repository (https://massive.ucsd.edu/ProteoSAFe/static/massive.jsp). The data can be accessed directly at GNPS/MassIVE under the accession number MSV000094551. The strains described in this study are available upon request. The strains PH060523057 (*Extibacter* sp.) and PH060523065 (*Megasphaera* sp.) are available in the international collections of the Pasteur Institute (CIP112496T and CIP 112498 T, respectively) and the Deutsche Sammlung von Mikroorganismen und Zellkulturen (DSM119879 and DSM119886, respectively). For further information or to obtain the strains, please contact the corresponding author at t.soranzo@pelican-health.com.
