# Supplementary material for: Exploring the human small intestinal luminal microbiome via a newly developed ingestible sampling device
Source: ISME Commun. 2025 Nov 28;5(1):ycaf224. doi: 10.1093/ismeco/ycaf224 (PMC12721380; doi:10.1093/ismeco/ycaf224)
Supplement: Supplementary_Figures_ISME_Comm_FINAL_ycaf224 [file supplementary_figures_isme_comm_final_ycaf224.docx]

**Supplementary Figures:**


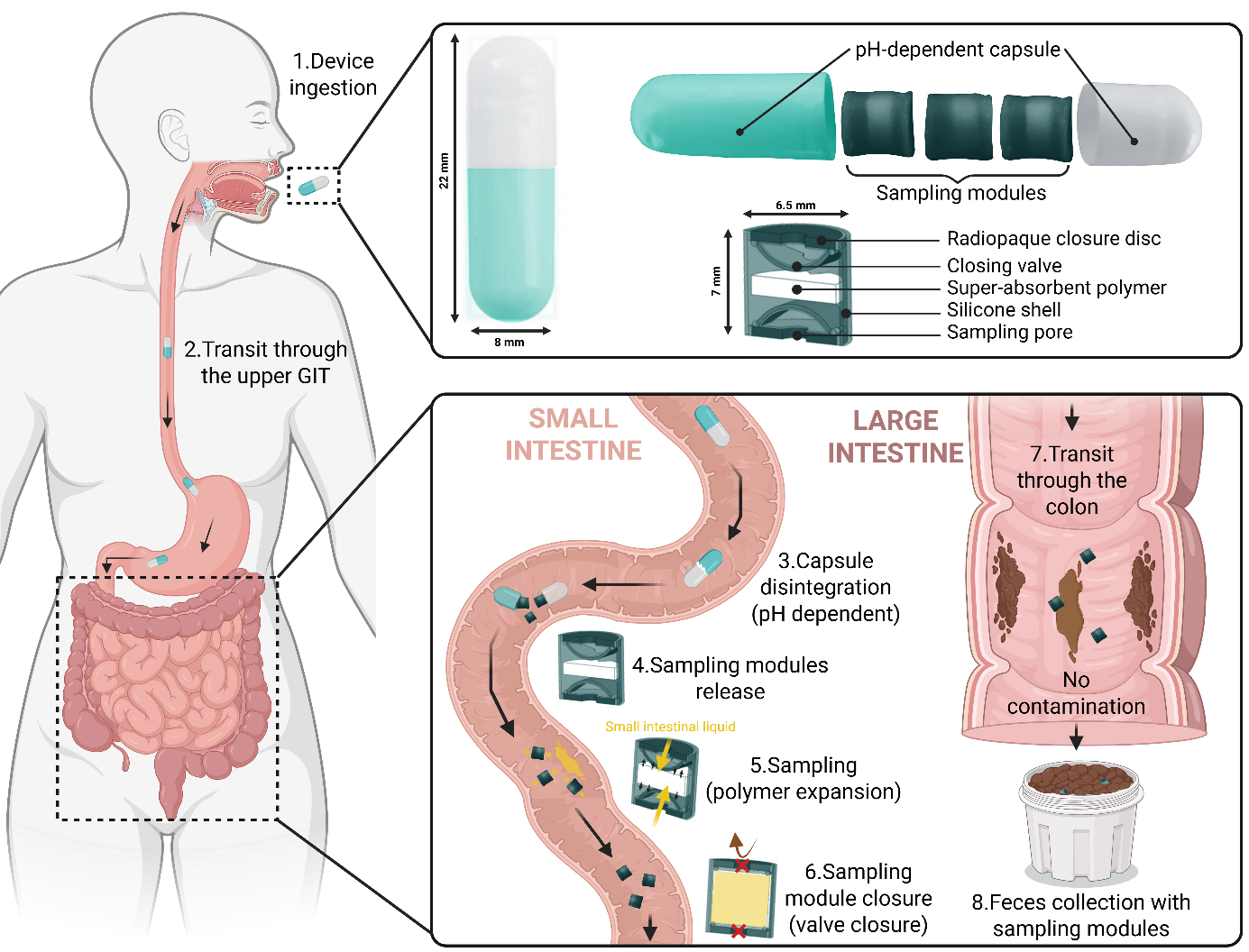


**Supplementary figure 1:** Schematic representation of the working principle of the medical device tested in the clinical investigation (figure created in BioRender).


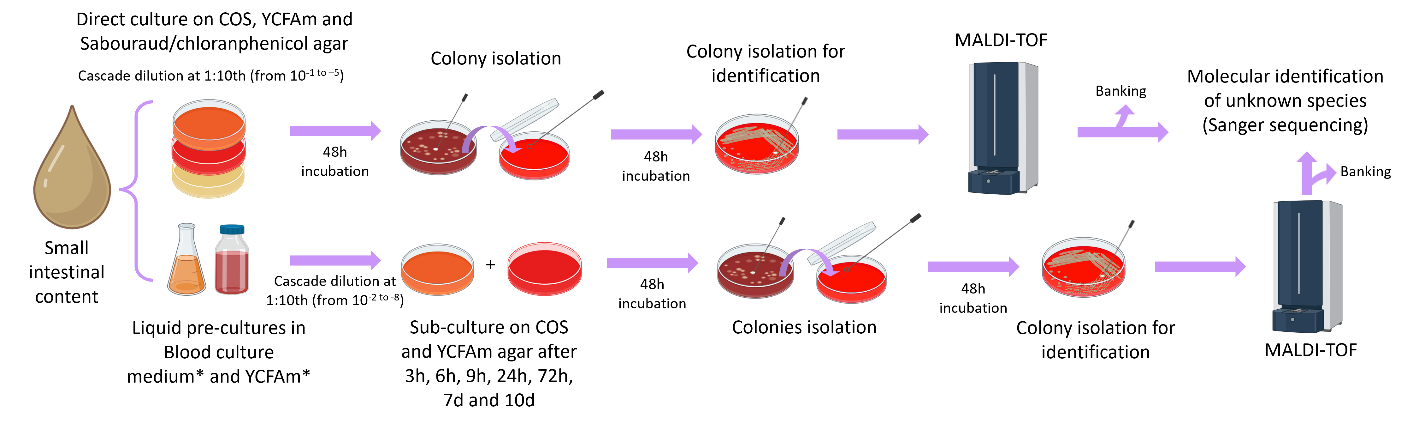


**Supplementary figure 2:** Culturomics workflow in anaerobic and aerobic conditions performed on small intestinal contents (figure created in BioRender)

**
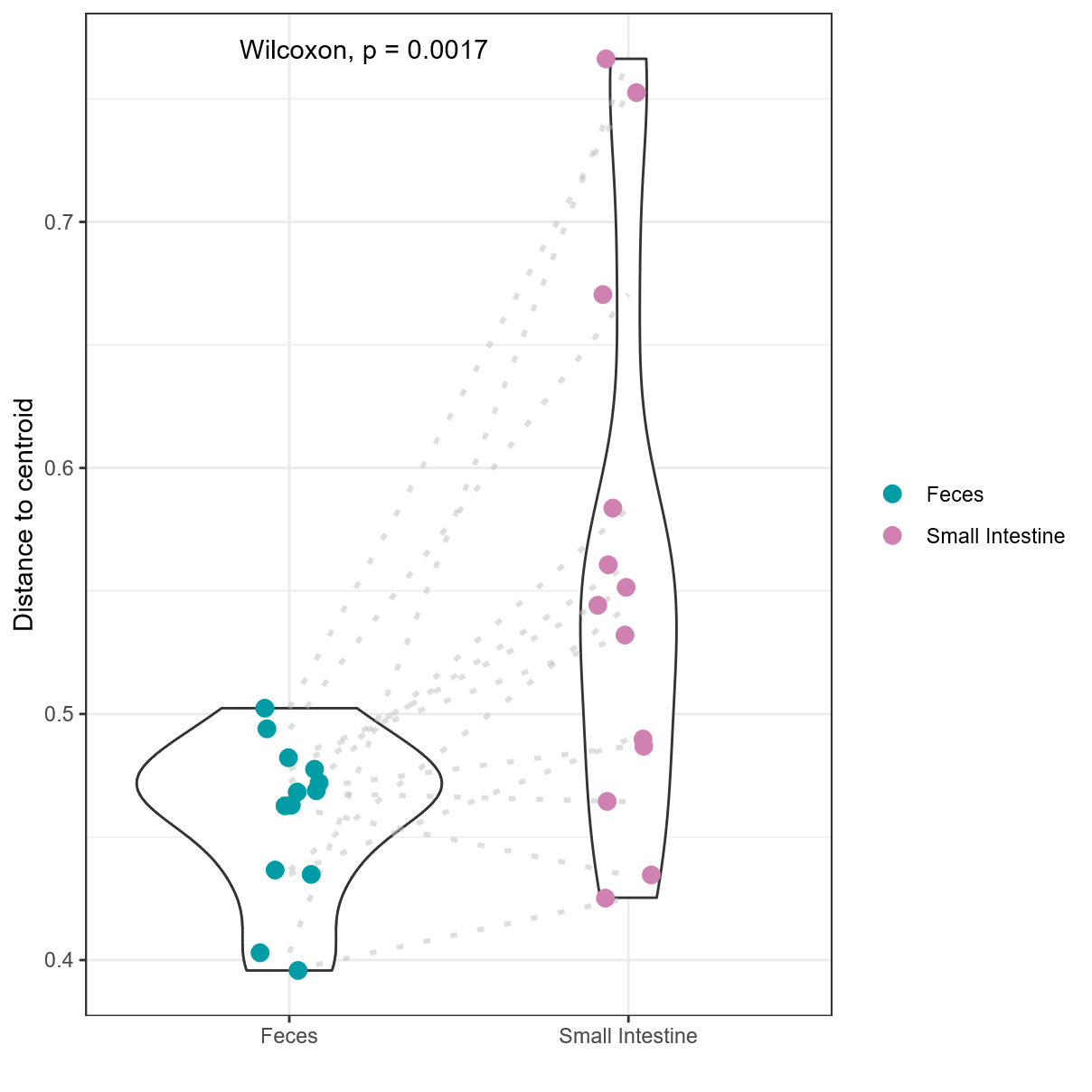
**

**Supplementary figure 3:** Permutational Analysis of Multivariate Dispersions (distance to the centroid) of small intestinal contents and feces using the PERMDISP function (R; vegan package). Differences in beta diversity among different areas or groups of samples were tested and results show a significant difference between the microbiota of the feces and SI samples.


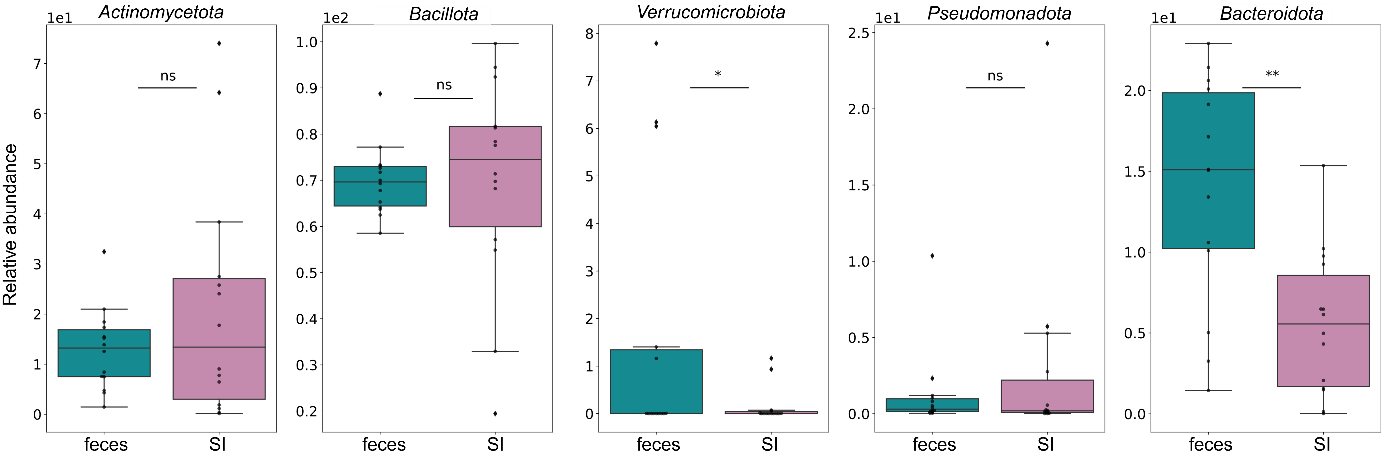


**Supplementary Figure 4:** Boxplots of the relative abundances at phylum level between the sample types (SI contents: n = 14; feces: n = 14). Wilcoxon test with adjusted p-value by using the Benjamini-Hochberg procedure with the False Discovery Rate (FDR). *: 0.01 < p-value < 0.05, **: 0.001 < p-value < 0.01, ns: non-significant.

**
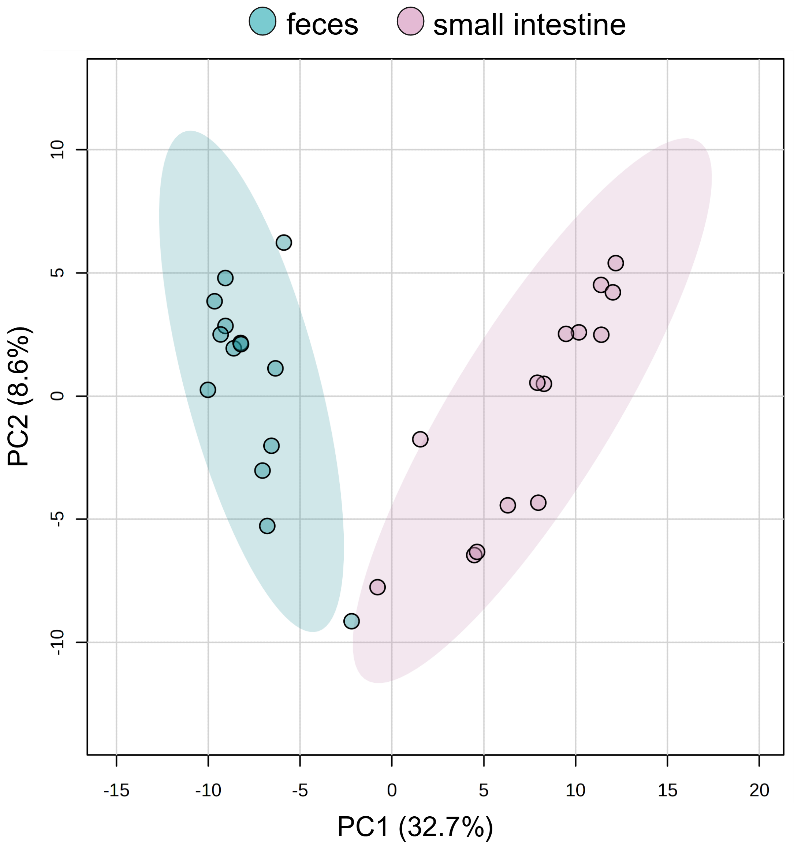
**

**Supplementary Figure 5:** Principal Component Analysis (PCA) of the filtered features (*n*=217 features) obtained in negative ionization mode. To assess statistical differences between the metabolomics profiles, PERMANOVA based on 999 Monte Carlo permutations was performed (F-value: 56.805; R-squared: 0.68601; p-value permutations: 0.001).


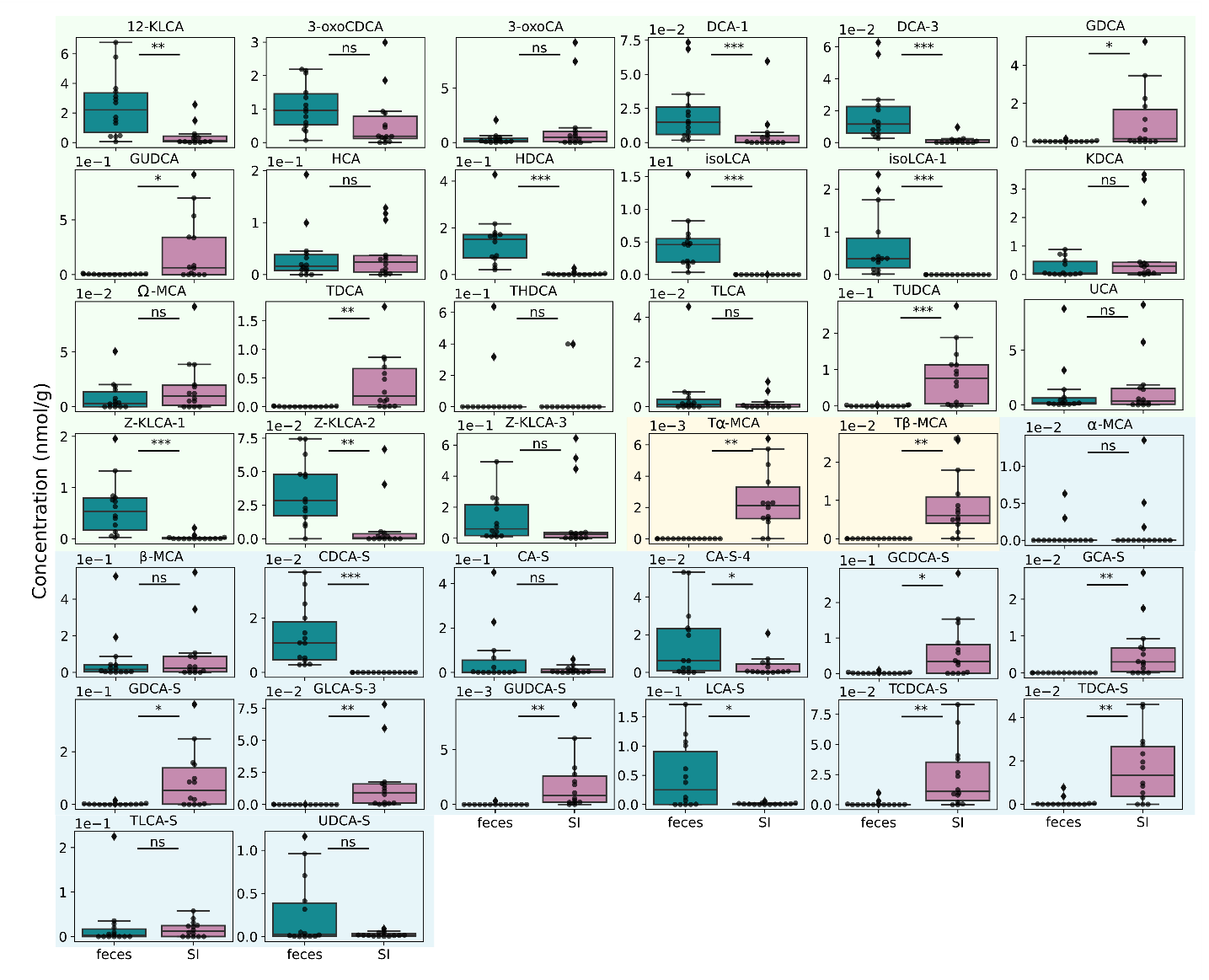


**Supplementary figure 6:** Boxplots of the quantified BAs in SI content (n = 14) versus feces (n = 14). Green: microbe-derived BAs, yellow: host-derived BAs and blue: host/microbe-derived BAs. 12-KLCA: 12-Ketolithocholic acid; 3-oxoCDCA: 3-Oxochenodeoxycholic acid; 3-oxoCA: 3-Oxocholic acid; DCA-1: Deoxycholic acid like 1; DCA-3: Deoxycholic acid like 3; GDCA: Glycodeoxycholic acid; GUDCA: Glycoursodeoxycholic acid; HCA: Hyocholic acid; HDCA: Hyodeoxycholic acid; isoLCA: Isolithocholic acid; isoLCA-1: Isolithocholic acid like; KDCA: Ketodeoxycholic acid like; Ω-MCA : omega-Muricholic acid; TDCA: Taurodeoxycholic acid; THDCA: Taurohyodeoxycholic acid; TLCA: Taurolithocholic acid; TUDCA: Tauroursodeoxycholic acid; UCA: Ursocholic acid; Z-KLCA-1: Z-Ketolithocholic acid-like 1; Z-KLCA-2: Z-Ketolithocholic acid-like 2; Z-KLCA-3: Z-Ketolithocholic acid-like 3; Tα-MCA: Tauro-alpha-Muricholic acid; Tꞵ-MCA: Tauro-beta-Muricholic acid; α-MCA: alpha-Muricholic acid; ꞵ-MCA: beta-Muricholic acid; CDCA-S: Chenodeoxycholic acid-sulfate like; CA-S: Cholic acid-sulfate like; CA-S-4: Cholic acid sulfate like 4; CDCA-S: Glycochenodeoxycholic acid-sulfate like; GCA-S: Glycocholic acid-sulfate-like; GDCA-S: Glycodeoxycholic acid-sulfate like; GLCA-S-3: Glycolithocholic acid-sulfate like 3; GUDCA-S: Glycoursodeoxycholic acid-sulfate like; LCA-S: Lithocholic acid-sulfate-like; TCDCA-S: Taurochenodeoxycholic acid-sulfate like; TDCA-S: Taurodeoxycholic acid-sulfate like; TLCA-S: Taurolithocholic acid sulfate and UDCA-S: Ursodeoxycholic acid-sulfate like. Taurocholic acid- sulfate like and glycolithocholic acid have not been represented because their concentrations were below the limit of detection in both sample types. Wilcoxon test with adjusted p-value by using the Benjamini-Hochberg procedure with the False Discovery Rate (FDR). *: 0.01 < p-value < 0.05, **: 0.001 < p-value < 0.01, ***: 0.0001 < p-value < 0.001, ns: non-significant.


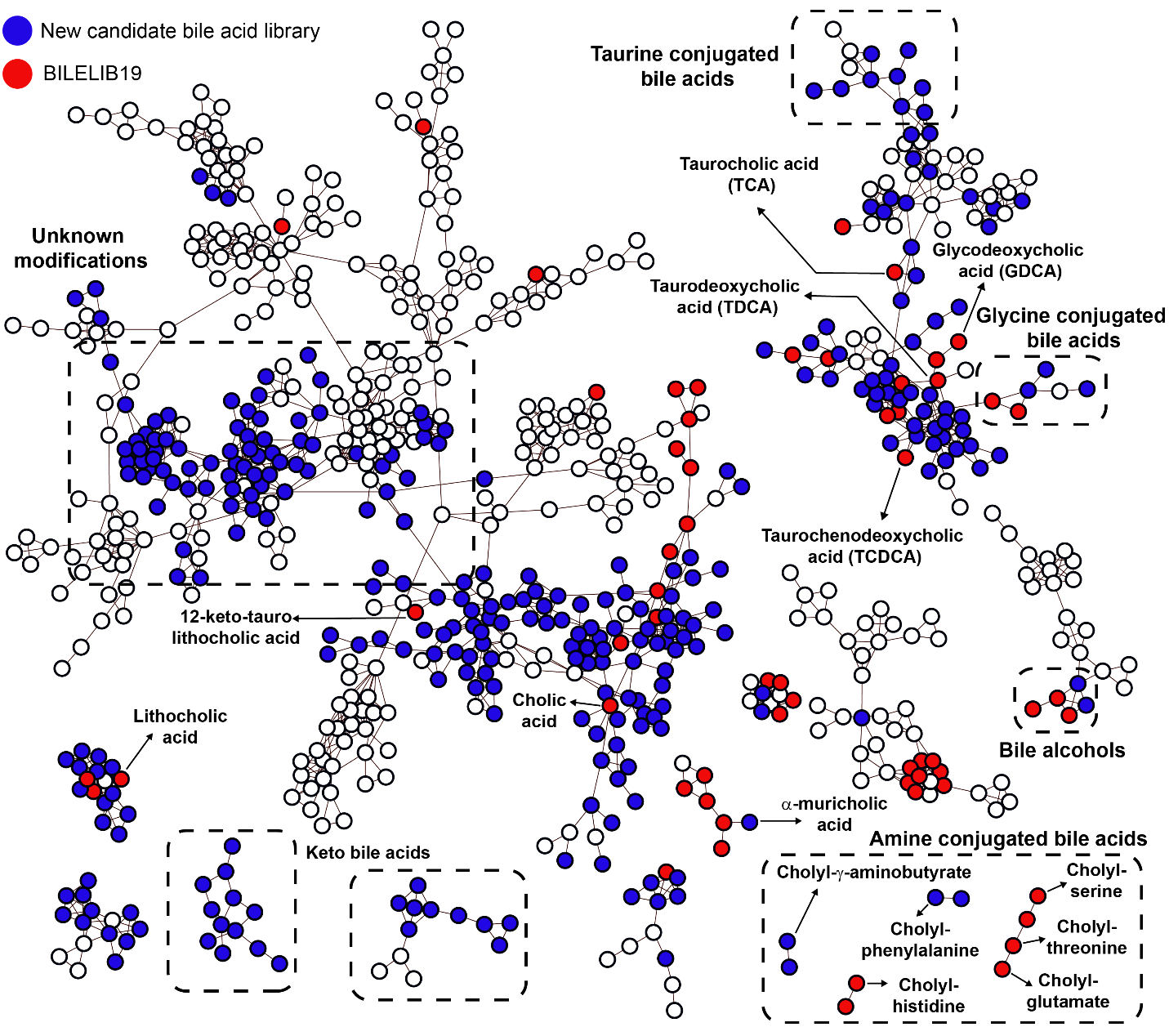


**Supplementary figure 7:** Feature-based molecular networking of annotated and non-annotated bile aids in fecal and SI samples of 14 patients. Molecular clusters containing spectral matches to two bile acid-specific MS2 libraries – the recently curated candidate library (blue nodes; 21,549 spectra are included in the library) and the BILELIB19 (red nodes; in total 5008 spectra are included in the library).
