# Supplementary material for: Exploring the human small intestinal luminal microbiome via a newly developed ingestible sampling device
Source: ISME Commun. 2025 Nov 28;5(1):ycaf224. doi: 10.1093/ismeco/ycaf224 (PMC12721380; doi:10.1093/ismeco/ycaf224)
Supplement: Supplementary_Methods_ISME_Comm_FINAL_ycaf224 [file supplementary_methods_isme_comm_final_ycaf224.pdf]

Supplementary methods

Determination of bacterial composition by metabarcoding sequencing

DNA extraction

For fecal samples, extraction was performed on 250 mg of sample. Conversely, DNA was extracted by an external service provider (ADM biopolis, Valencia, Spain) on the whole bacterial pellet retrieved from the SI content after centrifugation (15 min 15 000g). The protocol of the commercial PowerFecal Pro DNA Kit by Qiagen (Qiagen, Hilden, Germany) was used for DNA isolation. Briefly, a cell lysate was obtained by mechanical disruption (Fastprep, Thermo Fisher Scientific) and chemical treatment. The isolation of DNA and the purification of contaminants and inhibitors was performed using a silica column. For samples from intestinal content yielded very low concentration DNA, an extra step of purification was done following with QIAamp DNA Micro Kit (Qiagen, Hilden, Germany). Photometric (UV) evaluation was used to assess DNA quality and concentration using a Nanodrop apparatus (Thermo Fisher Scientific, Waltham, MA, USA).

16S rRNA sequencing analysis

The metabarcoding, sequencing and bioinformatic processing were carried out by an external service provider (ADM biopolis, Valencia, Spain). The DNA concentration used for amplification was 50 ng, following the 16S Metagenomic Sequencing Library Illumina 15044223 B protocol (Illumina). In summary, in the first amplification step, the region of interest has been amplified (V3-V4) with 16S rRNA gene universal primers [1] and a universal linker sequence allowing amplicons to incorporate indexes and sequencing primers during the secondary PCR step. The quantification of 16S-based amplicon was performed by fluorimetry using the Quant-iT™ PicoGreen™ dsDNA Assay Kit (Thermo fisher Scientific, Waltham, MA, USA). In the second assay amplification indexes Nextera XT Index kit (Illumina) were included. The quantification of 16S-based libraries was performed by fluorimetry using the Quant-iT™ PicoGreen™ dsDNA Assay Kit (Thermo fisher Scientific, Waltham, MA, USA). Libraries were pooled (equimolar). The size and quantity of the pool were assessed on the Bioanalyzer 2100 (Agilent) and with the Library Quantification Kit for Illumina (Kapa Biosciences), respectively. PhiX Control library (v3) (Illumina) was combined with the amplicon library (expected at 20%) prior to sequencing on the MiSeq platform (Illumina), 300 cycles in paired reads configuration. Image analyses, base calling, and data quality assessment were performed on the MiSeq instrument. Raw sequences, forward and reverse, were merged in order to obtain the paired-end sequences using the BBMerge package of BBMap V.38 software with minimum 70 nt overlap on each end. The amplification primers

from the sequences obtained in the sequencing step were trimmed to reduce bias in the annotation step, with Cutadapt v 1.8.1 and parameters by default. Once the primers were removed, sequences lower than 200 nt were removed from the analysis. After obtaining the clean complete sequences using Reformat module from BBMap v.38, a quality filter was applied to delete poor - quality sequences. Those bases in extreme positions that did not reach Q20 (99% well - incorporated base in the sequencing step) or a greater phred score were removed. Subsequently, sequences whose average quality did not surpass the Q20 threshold, as a mean quality of the whole sequence, were also deleted. The reads were processed using the DADA2[2] denoise-single command. Error rates were learned from a set of subsampled reads using "learnErrors" and a sample inference algorithm was applied with the "dada" function. The chimeric amplicon sequence variants (ASVs) were removed using "removeChimeraDenovo". Those clean ASVs were annotated against the NCBI 16S rRNA database version 2021 using Blastn version 2.2.29+ [3]. The taxonomy of the ASVs that had been assigned with a lower percentage identity than 97%, was reassigned using NBAYES algorithm [4]. NBAYES classifier was trained on V3-V4 regions of 16S rRNA gene from SILVA v.138 database [5]. Alpha- and beta-diversity analyses were performed in R using the phyloseq [6] and vegan packages. To account for differences in sequencing depth, all samples were normalized by rarefaction to the minimum read count of 46,557 reads per sample (excluding PBS controls). Richness was defined as the number of distinct ASVs, while Shannon and Simpson indices were used as composite measures of alpha diversity incorporating both richness and evenness. The Shannon index was calculated using the diversity function (vegan, method = "shannon"). Beta diversity was assessed using Bray–Curtis dissimilarities, followed by principal coordinate analysis (PCoA) and PERMDISP tests to evaluate group dispersion.

Determination of the metabolomes

Sample preparation for the untargeted and semi-targeted metabolomics

Two distinct samples types were prepared for Liquid Chromatography-High Resolution Mass Spectrometry tandem (LC-HRMS/MS) acquisition: the SI content sampled with the MD and the fecal samples. We approximated that the SI density is equivalent to the water density, resulting in: 1 µL = 1 mg.

For SI content, metabolites were extracted by the addition of cold methanol (MeOH) (1:4 (v/v)) spiked with deuterated leucine (d-leucine) for protein precipitation and then vortexed for 20s. For fecal samples, first the water content was determined by drying a part of the sample in an oven. Separately, a total of 150 mg of wet feces was used for metabolomic analyses. The water content was corrected by adding water to samples (correction based on the higher water content). Samples were then homogenized by vortex and sonication for 10 min (on ice). MeOH spiked with d-leucine was added (1:4

(w/v)) for metabolites extraction and protein precipitation. Fecal and intestinal samples were then incubated on ice for 30 min and centrifuged 15 min, 15 000 g, +4°C. After separation of supernatants and pellets, supernatants were evaporated under nitrogen flow at room temperature. The metabolite dry pellets were resuspended in liquid chromatographic solvent (80% water, 20% MeOH, 1% acetonitrile (ACN), 0,1% formic acid (FA) and internal standards (deuterated tryptophan and deuterated phenylalanine)). Resuspension volumes were different for each SI sample to normalize the concentration of intestinal fluid injected during the analysis.

Combined untargeted and semi-targeted metabolomics approach

A combined untargeted and semi-targeted metabolomics approach was applied using an Ultra-high-performance liquid chromatography (UHPLC) (Vanquish Flex, Thermo Fisher Scientific, Waltham, MA, USA) coupled with a Q Exactive Plus Orbitrap HRMS/MS (Thermo Fisher Scientific, Waltham, MA, USA) [7]. Thirty-seven metabolites of interest that have been previously selected [7] (listed in Supplementary Table 1), have been relatively quantified with the semi-targeted method.

The chromatographic separation was carried out on a Luna Omega polar C18 (2.1mm × 150 mm, 1.6 µm, Phenomenex, Torrance, CA, USA) at 30 °C with a flow elution rate of 400 µL/min. The temperature of the autosampler compartment was set at +4°C, and the injection volume was 5 µL. For the chromatographic settings, the mobile phases consisted of A (water + 0.1% FA) and B (ACN + 0.1% FA). Elution started with an isocratic step of 2 min at 1% mobile phase B, followed by a linear gradient from 1% to 100% mobile phase B for the next 12 min. The next 6 min were with 100% mobile phase B before returning to 1% B for 5 min corresponding to the column equilibration. The entire chromatographic acquisition lasted 25 min. The mass spectrometer was fitted with an electrospray source (ESI) operating in positive and negative ionization modes. The scan range was from *m/z* 85.0 to 1275.0 with a resolution of 70 000 at a ratio *m/z* = 200. MS2 fragmentation was performed at three collision energies (CEs: 10, 35, 55 eVs) on the top 10 of the most abundant parent ions during full scan. Quality controls (QC) were used to ensure the quality of the LC-HRMS/MS acquisition. Deuterated compounds were used for controlling extraction process (d-leucine) and injection (d-tryptophan and d-phenylalanine). Pooled QC assembled from all biological samples ensured the stability of peak detection and intensity. They were injected every 10 injections. Briefly, for the semi-targeted metabolomics, an external calibration curve with the pooled 37 metabolites of interest was injected at different dilution factor, allowing to estimate the concentrations of each metabolite in the biological samples [7].

Data were then analyzed for untargeted metabolomics MZmine 3.9.0 [8] as described elsewhere [9]. We evaluated the threshold's noise on our instrument in full scan at an intensity of 1E6 and 1E3 in MS2. After data processing on MZmine3, data was filtrated based on a coefficient of variation for each

feature on the QC pool lower than 30% and the feature must be present in 10% of the total of biological samples. Feature annotation was achieved using Feature Based Molecular Networking (FBMN) [10] on Global Natural Products Social Networking 2 (GNPS2).

The semi-targeted analysis was performed using the Thermo Fisher Scientific software TraceFinder 4.1 General Quan and has been previously described [7]. Briefly, after the construction of the compound database containing the 37 metabolites of interest, samples were analyzed by the software to find the metabolites based on full scan spectra, retention time and MS2 spectra. Data containing peak areas for each sample were then exported in .csv file for deeper analysis. Precisely, sample losses due to the metabolite extraction method were assessed by measuring the recovery percentage by dividing the detected d-leucine in each sample by the expected d-leucine quantity. Then the following formula is applied to obtain metabolite quantity in arbitrary unit per mg for both sample types: peak area / (recovery*mg). These data were plotted to obtain boxplots for feces or intestinal liquid for each metabolite with Matplotlib v. 3.5.1 [11] in Python v. 3.9.5.

To detect as many BAs as possible, we reprocessed the untargeted metabolomics data by reducing filtration. BA annotations and relative quantification using peak area abundances extracted using MZmine 4.1.0 [8] were achieved using FBMN [10] on GNPS2 and an expanded set of bile acid libraries [12,13].

Sample preparation for the targeted metabolomics on bile acids

A fraction of the samples from feces and SI content were mixed with MeOH (4:1) and homogenized with a bead beater for 4 min. Subsequently, they were centrifuged at 16 000 g at 4°C for 10 min. The supernatant was then transferred to Phree filters and centrifuged at 15 000 g at 4°C for 5 min. The filtrates were stored at -20 °C until analysis.

Targeted metabolomics analysis on bile acids

The analysis of BAs was performed by an external provider (Clinical Microbiomics, Vedbæk, Denmark) and was carried out in a randomized order using a UHPLC system (Vanquish, ThermoFisher Scientific, Waltham, MA, USA) coupled with a high-resolution mass spectrometer Q Exactive™ HF Hybrid Quadrupole-Orbitrap, (Thermo Fisher Scientific, Waltham, MA, USA). An electrospray ionization interface was used as an ion source in negative mode. The chromatographic separation of bile acids was carried out on a Waters Acquity HSS T3 1.8 µm 2.1 x 150 mm (Waters). The column was thermostated at 30°C. The mobile phases consisted of (A) ammonium acetate 10 mM, and (B) MeOH/ACN (1:1, v/v). Bile acids were eluted by increasing B in A from 45 to 100 % for 16 min. Flow rate was 0.3 ml/min. To ensure high-quality sample preparation, a quality control sample (QC sample)

was prepared by pooling small equal aliquots from each sample, to create a representative average of the entire set. This sample was treated and analyzed at regular intervals throughout the sequence. Possible matrix effects on compounds for quantification, were tested by spiking aliquots of the QC sample at a minimum of two levels. A QC sample was analyzed in MS/MS mode for the identification of compounds. Peak areas were extracted using Skyline 23.1 (MacCoss Lab Software). Identification of compounds was based on accurate mass and retention time of authentic standards. The concentrations of 50 BAs have been determined. The BA list is available in Supplementary Table 2.

Determination of microbial composition by a fast culturomics approach

Media preparation

Different media were used for fast culturomics. The first one is Yeast Casitone Fatty Acids (YCFA) medium (Deutsche Sammlung von Mikroorganismen und Zellkulturen, DSMZ_medium1611). Liquid YCFA was degassed with argon gas for 15 min and autoclaved before filling into Hungate tubes (10 mL) and supplemented with 2mL of anaerobic sterile rumen juice and 2 mL of anaerobic sterile defibrinated sheep blood (SARL Atlantis, Voulmentin, France). For solid YCFA, the medium was enriched with 5% of sterile rumen juice and 5% of defibrinated sheep blood. Sterile rumen juice was prepared following the procedure described by Diakite *et al.*[14]. The other solid media were sheep blood agar plate (COS) (BioMérieux, Marcy l'Etoile, France) and Sabouraud/Chloramphenicol agar plate (BioMérieux, Marcy l'Etoile, France). In addition, two other liquid media were used: anaerobic culture vial medium and aerobic culture vial medium from BD BACTEC™ (Becton Dickinson, Franklin Lakes, NJ, USA). These media were supplemented with 2 mL of sterile rumen juice and 2 mL of defibrinated sheep blood.

Fast culturomics protocol

Culturomics was performed on two intestinal samples just after the module's collection in feces. To be used for culturomics, intestinal samples must meet criteria such as have a sufficient collected volume >50µL and no fecal contamination (dark spot on the polymer).

The fast culturomics protocol was adapted from the fast culturomics method developed by Naud *et al.* [15]. In summary, the small intestinal content was placed immediately after collection in an anaerobic bag and culturomics process started within one hour. Following gentle centrifugation and phosphate buffer saline (PBS) washes (two cycles) (at the conclusion of which the sample was 10-fold diluted), the intestinal suspension was placed in an anaerobic chamber (Whitley A35 anaerobic station, Don Whitley scientific, Bingley, England). The gas alimenting the anaerobic chamber was composed of 90% nitrogen, 5% hydrogen and 5% carbon dioxide. The experimental design incorporated a range of culture conditions. First, 100 µL of the 10 times diluted sample are used for a series of dilutions (from 10^{-1} to

10^{-5}) in PBS and 50 µL of each dilution are plated on YFCA modified, COS and Sabouraud/Chloramphenicol agar plates. These direct cultures were then subjected to incubation for a period of 48 hours at a temperature of 37°C, within either an anaerobic or an aerobic atmosphere. Concurrently, pre-cultures (liquid enrichment) have been performed in YCFA-modified liquid medium and anaerobic/aerobic culture vial media. These pre-cultures were then subjected to incubation at 37°C within either anaerobic or aerobic atmospheres without agitation. Sub-culturing was performed after three hours, six hours, nine hours, 24 hours, 72 hours, seven days, and 10 days of incubation. To this end, a dilution series ranging from 10^{-2} to 10^{-8} was established using 100 µL of the pre-culture. These dilutions (50 µL) were subsequently plated onto YCFA agar plates and COS agar plates, with the former being used for the YCFA pre-culture and the latter for the anaerobic/aerobic culture vial media. These plates were then subjected to an incubation period of 48 hours at a temperature of 37°C within either an anaerobic or an aerobic atmosphere.

Microbial identification

The isolation of microbial colonies was conducted following a 48-hour incubation period, contingent on their distinct morphological characteristics, including size and color. Isolates were then streaked again on the same agar plate from which they were initially isolated (YCFA or COS). Following an additional 48 hours of incubation, identification of the isolates was conducted through the use of mass spectrometry Matrix Assisted Laser Desorption-Time of Flight (MALDI-TOF). The MALDI-TOF MS Biotyper (Bruker Daltonik, Bremen, Germany) was utilized for this purpose. The obtained spectra were then compared to the MBT IVD Library Revision J (2022), which contains spectra from 4,194 microbial species. Colony identifications were considered valid if the identification score was ≥1.8. In instances where the identification score was less than 1.8, a secondary MALDI-TOF analysis was conducted to ascertain the accuracy of the initial identification. If the score fell below 1.8 on the second attempt, the isolate was designated as "unknown."

The identification of unknown isolates necessitated the implementation of full 16S rRNA gene sequencing. Genomic DNA was extracted using the PowerFecal Pro DNA Kit by Qiagen (Qiagen, Hilden, Germany). Full 16S rRNA gene DNA was amplified by PCR using the following primers: F: 5’-AGRGTTYGATYMTGGCTCAG and R: 5’-CGGYTACCTTGTTACGACTT and the KAPA2G Robust HotStart ReadyMix (Kapa Biosystems, Wilmington, MA, USA). The PCR conditions were as follows: initial denaturation at 95°C for 3 minutes, denaturation at 95°C for 15 seconds, annealing at 60°C for 15 seconds, extension at 72°C for 60 seconds and final extension at 72°C for 2 minutes. A total of 30 cycles were completed. Subsequently to this, the amplicons were then purified using the magnetic beads purification kit AMPure, and the DNA was quantified with a Nanodrop (Thermo Fisher Scientific,

Waltham, MA, USA). Subsequently, Sanger sequencing was performed using the same primers as the PCR. The QV30 sequences were then subjected to analysis with the aid of the Benchling software, wherein the forward and reverse sequences were aligned to construct the consensus sequence (alignment program MAFFT [16]). The consensus sequences were then analyzed using EZbiocloud [17]. The definition of a new species was based on a 16S rRNA gene sequence similarity of less than 98.65% with the closest relatives [18].

Isolates were banked in YCFA or anaerobic culture vial media and 20% glycerol and stored at -80°C.

Statistical analysis

All statistical analyses were performed using R software with *vegan* [19] package for diversity analysis and *phyloseq* [20] for microbiome data handling. Alpha diversity was evaluated as species richness, defined as the number of different ASVs, and species diversity via the Shannon index, which integrates richness and evenness. Statistical comparisons were conducted using a paired Wilcoxon rank-sum test, with a p-value threshold set at < 0.05. The beta diversity (differences in microbial composition) between SI content and fecal samples was evaluated using Bray-Curtis dissimilarity matrix and visualized via Principal Coordinate Analysis (PCoA). The statistical significance of the observed differences in the PCoA was evaluated using PERMANOVA (Permutational Multivariate Analysis of Variance) with 999 permutations, and a p-value threshold of < 0.05. To assess the variability and interindividual variation within groups PERMDISP (Permutational Analysis of Multivariate Dispersions) was used to evaluate the homogeneity of group dispersions. This was achieved by calculating the distance of each sample to the group centroid and comparing these distances between groups.

In order to ascertain the bacterial genera that potentially serve as biomarkers of biological matrices, the fold change (FC) (SI+1/feces+1) was determined for each bacterial genus from the normalized relative abundance data. The analysis was conducted on genera present in a minimum of 20% of the intestinal content or fecal samples. The Wilcoxon statistical test was employed to compare the mean relative abundances between the two groups. P-values were adjusted by using the Benjamini-Hochberg procedure with the False Discovery Rate (FDR).

For the untargeted metabolomic approach, MetaboAnalyst 6.0 [21] was utilized. Normalization of samples was achieved through median normalization, and data were log-transformed. To ensure data integrity and enhance interpretability, Pareto scaling was employed for data scaling. Volcano plots were generated using MetaboAnalyst 6.0, and the top 30 features were annotated on the plot. The selected FC (small intestine/feces) threshold was 2, and the p-value threshold was 0.05.

For semi-targeted and BAs targeted metabolomics, statistical analysis was applied to assess significant differences between feces and intestinal content thanks to a non-parametric Wilcoxon test. In this case, p-values were corrected by using the Benjamini-Hochberg procedure with the False Discovery Rate (FDR) obtained.

References

1. Klindworth A, Pruesse E, Schweer T, Peplies J, Quast C, Horn M, et al. Evaluation of general 16S ribosomal RNA gene PCR primers for classical and next-generation sequencing-based diversity studies. Nucleic Acids Research. 2013;41:e1. https://doi.org/10.1093/nar/gks808

2. Callahan BJ, McMurdie PJ, Rosen MJ, Han AW, Johnson AJA, Holmes SP. DADA2: High-resolution sample inference from Illumina amplicon data. Nat Methods. Nature Publishing Group; 2016;13:581–3. https://doi.org/10.1038/nmeth.3869

3. Altschul SF, Gish W, Miller W, Myers EW, Lipman DJ. Basic local alignment search tool. J Mol Biol. 1990;215:403–10. https://doi.org/10.1016/S0022-2836(05)80360-2

4. Bokulich NA, Kaehler BD, Rideout JR, Dillon M, Bolyen E, Knight R, et al. Optimizing taxonomic classification of marker-gene amplicon sequences with QIIME 2's q2-feature-classifier plugin. Microbiome. 2018;6:90. https://doi.org/10.1186/s40168-018-0470-z

5. Quast C, Pruesse E, Yilmaz P, Gerken J, Schweer T, Yarza P, et al. The SILVA ribosomal RNA gene database project: improved data processing and web-based tools. Nucleic Acids Res. 2013;41:D590–6. https://doi.org/10.1093/nar/gks1219

6. Weiss S, Xu ZZ, Peddada S, Amir A, Bittinger K, Gonzalez A, et al. Normalization and microbial differential abundance strategies depend upon data characteristics. Microbiome. 2017;5:27. https://doi.org/10.1186/s40168-017-0237-y

7. Tronel A, Roger-Margueritat M, Plazy C, Cunin V, Mohanty I, Dorrestein PC, et al. Untargeted and Semi-Targeted Metabolomics Approach for Profiling Small Intestinal and Fecal Metabolome Using High-Resolution Mass Spectrometry [Internet]. bioRxiv; 2024 [cited 2024 Sep 23]. p. 2024.09.16.613180. https://doi.org/10.1101/2024.09.16.613180

8. Schmid R, Heuckeroth S, Korf A, Smirnov A, Myers O, Dyrlund TS, et al. Integrative analysis of multimodal mass spectrometry data in MZmine 3. Nat Biotechnol. Nature Publishing Group; 2023;41:447–9. https://doi.org/10.1038/s41587-023-01690-2

9. Damiani T, Heuckeroth S, Smirnov A, Mokshyna O, Brungs C, Korf A, et al. Mass spectrometry data processing in MZmine 3: feature detection and annotation [Internet]. ChemRxiv; 2023 [cited 2024 Jul 22]. https://doi.org/10.26434/chemrxiv-2023-98n6q

10. Nothias L-F, Petras D, Schmid R, Dührkop K, Rainer J, Sarvepalli A, et al. Feature-based molecular networking in the GNPS analysis environment. Nat Methods. Nature Publishing Group; 2020;17:905–8. https://doi.org/10.1038/s41592-020-0933-6

11. Hunter JD. Matplotlib: A 2D Graphics Environment. Computing in Science & Engineering. 2007;9:90–5. https://doi.org/10.1109/MCSE.2007.55

12. Mohanty I, Mannochio-Russo H, Schweer JV, Abiead YE, Bittremieux W, Xing S, et al. The underappreciated diversity of bile acid modifications. Cell [Internet]. Elsevier; 2024 [cited 2024 Mar 21];0. https://doi.org/10.1016/j.cell.2024.02.019

13. Gentry EC, Collins SL, Panitchpakdi M, Belda-Ferre P, Stewart AK, Carrillo Terrazas M, et al. Reverse metabolomics for the discovery of chemical structures from humans. Nature. Nature Publishing Group; 2024;626:419–26. https://doi.org/10.1038/s41586-023-06906-8

14. Diakite A, Dubourg G, Dione N, Afouda P, Bellali S, Ngom II, et al. Optimization and standardization of the culturomics technique for human microbiome exploration. Sci Rep. 2020;10:9674. https://doi.org/10.1038/s41598-020-66738-8

15. Naud S, Khelaifia S, Mbogning Fonkou MD, Dione N, Lagier J-C, Raoult D. Proof of Concept of Culturomics Use of Time of Care. Frontiers in Cellular and Infection Microbiology [Internet]. 2020 [cited 2022 Jun 2];10. https://www.frontiersin.org/article/10.3389/fcimb.2020.524769. Accessed 2 Jun 2022

16. Katoh K, Standley DM. MAFFT Multiple Sequence Alignment Software Version 7: Improvements in Performance and Usability. Molecular Biology and Evolution. 2013;30:772–80. https://doi.org/10.1093/molbev/mst010

17. Chalita M, Kim YO, Park S, Oh H-S, Cho JH, Moon J, et al. EzBioCloud: a genome-driven database and platform for microbiome identification and discovery. Int J Syst Evol Microbiol. 2024;74. https://doi.org/10.1099/ijsem.0.006421

18. Kim M, Oh H-S, Park S-C, Chun J. Towards a taxonomic coherence between average nucleotide identity and 16S rRNA gene sequence similarity for species demarcation of prokaryotes. Int J Syst Evol Microbiol. 2014;64:346–51. https://doi.org/10.1099/ijs.0.059774-0

19. Dixon P. VEGAN, a package of R functions for community ecology. Journal of Vegetation Science. 2003;14:927–30. https://doi.org/10.1111/j.1654-1103.2003.tb02228.x

20. McMurdie PJ, Holmes S. phyloseq: An R Package for Reproducible Interactive Analysis and Graphics of Microbiome Census Data. PLOS ONE. Public Library of Science; 2013;8:e61217. https://doi.org/10.1371/journal.pone.0061217

21. Pang Z, Lu Y, Zhou G, Hui F, Xu L, Viau C, et al. MetaboAnalyst 6.0: towards a unified platform for metabolomics data processing, analysis and interpretation. Nucleic Acids Research. 2024;52:W398–406. https://doi.org/10.1093/nar/gkae253
