# Supplementary material for: Exploring the human small intestinal luminal microbiome via a newly developed ingestible sampling device
Source: ISME Commun. 2025 Nov 28;5(1):ycaf224. doi: 10.1093/ismeco/ycaf224 (PMC12721380; doi:10.1093/ismeco/ycaf224)
Supplement: Supplementary_table_9_FINAL_ycaf224 [file supplementary_table_9_final_ycaf224.docx]

**Supplementary table 9: Bacterial composition of small intestinal samples collected using different methods**

|  | **Cohorts** | **Sample type** | **Methods** | **Small intestinal microbiota structure compared to feces** | |
| --- | --- | --- | --- | --- | --- |
|  |  |  |  | **Diversity** | **Microbial composition** |
| Current study | Healthy subjects  (n=14) | Sampling capsule | 16S rRNA amplicons | ↓ α-diversity | ↑ *Streptococcus*, *Gemella*, *Granulicatella* (*Bacillota*) ↓ *Bacteroides*  ↑ *Actinomycetota* (*Bifidobacterium*, *Schaalia*) ↓ *Verrucomicrobiota* |
| [1] | Healthy subjects  (n=15) | Sampling capsule | 16S rRNA amplicons | ↓ α-diversity | ↑ *Pseudomonadota* (*Escherichia*/*Shigella*)  ↑ *Enterococcus, Bacteroides and Romboutsia* |
| [2] | Healthy subjects  (n=20) | Sampling capsule | 16S rRNA amplicons | ↓ α-diversity | ↑ *Streptococcus, Veillonella, Actinomyces, Gemella* and TM7x |
| [3] | Healthy subjects  (n=20) | Catheter aspiration | 16S rRNA amplicons | ↓ α-diversity | ↑ *Streptococcaceae, Prevotellaceae, Veillonellaceae, Pasteurellaceae* and *Enterobacteriaceae*. |
| [4] | Healthy subjects  (n=8) | Catheter aspiration | 16S rRNA amplicons | ↓ α-diversity | ↑ *Bacillota* (*Streptococcus, Veillonella, Gemella*)  ↑ *Prevotella* |
| [5] | Cadavers*  (n=33) | Cadaver | 16S rRNA amplicons | ↓ α-diversity | ↑ *Bacillota*  ↑ *Pseudomonadota*  Different composition between luminal and mucosal samples |
| [6] | Healthy Subjects  (n=25) | Endoscopy | 16S rRNA amplicons | - | ↑ *Prevotella, Streptococcus, Veillonella, Neisseriaceae* |
| [7] | CRC  (n=79) | Ileostomy | 16S rRNA amplicons | ↓ α-diversity | ↑ *Bacilli* and *Enterobacteriaceae*  ↑ *Lactobacillus, Clostridium, Streptococcus, Enterococcus* and *Veillonella*  ↓ *Ruminococcus* and *Bacteroides* |
| [8] | Patients**  (n=30) | Ileostomy | 16S rRNA amplicons | ↓ α-diversity | ↑ *Escherichia*/*Shigella* and *Streptococcus* |
| [9] | Healthy ileostomists  (n=5) | Ileostomy | Microarray HITChip | ↓ α-diversity | ↑ *Bacteroidota, Clostridium cluster XIVa and Pseudomonadota, Streptococcus, Veillonella* |
| [10] | SBT patients  (n=9) | Ileostomy | 16S rRNA amplicons | - | ↑ *Lactobacillales, Enterobacteriales, Bacteroidales and Clostridiales* |

CRC: Colorectal cancer, SBT: Small Bowel Transplantation, * who were dead due to vehicle accident, high-altitude falling, etc. To minimize the post-mortem microbial changes, all samples were collected in a short duration (<1.5 h) after determination of death. **who underwent laparoscopic anterior resection of the rectum with ileostomy, followed by ileostomy closure.

1. Shalon D, Culver RN, Grembi JA, Folz J, Treit PV, Shi H, et al. Profiling the human intestinal environment under physiological conditions. Nature. Nature Publishing Group; 2023;1–11. https://doi.org/10.1038/s41586-023-05989-7

2. Wang G, Menon S, Wilsack L, Rehak R, Lou L, Turbide C, et al. Spatially and Temporally Precise Microbiome Profiling in the Small Intestine using the SIMBA Capsule with X-ray tracking [Internet]. medRxiv; 2024 [cited 2024 Apr 12]. p. 2024.04.02.24305212. https://doi.org/10.1101/2024.04.02.24305212

3. An R, Wilms E, Gerritsen J, Kim HK, Pérez CS, Besseling-van der Vaart I, et al. Spatio-temporal dynamics of the human small intestinal microbiome and its response to a synbiotic. Gut Microbes. Taylor & Francis; 2024;16:2350173. https://doi.org/10.1080/19490976.2024.2350173

4. Seekatz AM, Schnizlein MK, Koenigsknecht MJ, Baker JR, Hasler WL, Bleske BE, et al. Spatial and Temporal Analysis of the Stomach and Small-Intestinal Microbiota in Fasted Healthy Humans. mSphere [Internet]. American Society for Microbiology; 2019 [cited 2022 Feb 21]; https://doi.org/10.1128/mSphere.00126-19

5. She J-J, Liu W-X, Ding X-M, Guo G, Han J, Shi F-Y, et al. Defining the biogeographical map and potential bacterial translocation of microbiome in human ‘surface organs.’ Nat Commun. Nature Publishing Group; 2024;15:427. https://doi.org/10.1038/s41467-024-44720-6

6. Zmora N, Zilberman-Schapira G, Suez J, Mor U, Dori-Bachash M, Bashiardes S, et al. Personalized Gut Mucosal Colonization Resistance to Empiric Probiotics Is Associated with Unique Host and Microbiome Features. Cell. 2018;174:1388-1405.e21. https://doi.org/10.1016/j.cell.2018.08.041

7. Yilmaz B, Fuhrer T, Morgenthaler D, Krupka N, Wang D, Spari D, et al. Plasticity of the adult human small intestinal stoma microbiota. Cell Host & Microbe. 2022;30:1773-1787.e6. https://doi.org/10.1016/j.chom.2022.10.002

8. Xu L, Li X, Chen L, Ma H, Wang Y, Liu W, et al. Gut microbiome and plasma metabolome alterations in ileostomy and after closure of ileostomy. Microbiology Spectrum. American Society for Microbiology; 2025;13:e01191-24. https://doi.org/10.1128/spectrum.01191-24

9. Zoetendal EG, Raes J, van den Bogert B, Arumugam M, Booijink CC, Troost FJ, et al. The human small intestinal microbiota is driven by rapid uptake and conversion of simple carbohydrates. ISME J. 2012;6:1415–26. https://doi.org/10.1038/ismej.2011.212

10. Hartman AL, Lough DM, Barupal DK, Fiehn O, Fishbein T, Zasloff M, et al. Human gut microbiome adopts an alternative state following small bowel transplantation. Proceedings of the National Academy of Sciences. Proceedings of the National Academy of Sciences; 2009;106:17187–92. https://doi.org/10.1073/pnas.0904847106
